# Long-term homogenized air temperature and precipitation datasets in Romania, 1901–2023

**DOI:** 10.1038/s41597-025-05371-4

**Published:** 2025-07-01

**Authors:** Alexandru Dumitrescu, Dana Micu, Jose Guijarro, Ancuta Manea, Sorin Cheval

**Affiliations:** 1 Meteo Romania (National Meteorological Administration), Bucharest, Romania; 2 Retired from State Meteorological Agency (AEMET), Balearic Islands Office, Palma de Mallorca, Spain; 3 Doctoral School of Geography, Faculty of Geography, Babeș-Bolyai University, Cluj-Napoca, Romania

**Keywords:** Climate change, Research data

## Abstract

In this study, we present RoClimHom, the first long-term homogenized dataset of daily air temperature and precipitation measurements for Romania, comprising mean (Tavg), maximum (Tmax), and minimum (Tmin) temperatures at 2 m height (T2m) and precipitation (PREC) from 156 weather stations spanning the 1901–2023 period. This work addresses the historical inconsistencies caused by changes in measurement techniques, station relocations, and operational practices. The homogenization methodology involved multiple stages, including data preprocessing, quality control, and breakpoint detection, resulting in a robust dataset that eliminates non-climatic biases. RoClimHom overcomes the potential biases in air temperature and precipitation measurements induced by changes in location, procedures, instruments, or personnel of the weather stations, which commonly influence natural climate variability. The new homogenized and gap-free climate dataset serves as an essential resource for academia and policymakers, facilitating climate change assessment and supporting a wide range of climate applications.

## Background & Summary

### Context and motivation

Based on homogenized data series, sound knowledge about the observed climate variability over different time scales is crucial for detecting trends and constructing reliable climate projections. The long-term variability is currently well-documented over large areas of Europe and North America, taking benefits of centennial meteorological records^1^ or at least consistent coverage of the 20th century^2–5^. Numerous relevant products are available, consisting of long-term, spatially contiguous, and temporally consistent gridded meteorological datasets. These datasets are invaluable for understanding climate variability and change. They are widely applied in areas such as climate trend and extremes analysis, statistical downscaling, and hydrological modeling for climatological studies. Examples include global coverage and widely used climate datasets such as the Global Precipitation Climatology Centre dataset (GPCCD)^6^, Integrated Surface Database (ISD)^7^, Berkley Earth Land/Ocean Temperature^8^ dataset or the Climatic Research Unit Timeseries (CRU TS)^9^. Other notable examples include continental or regional scale datasets such as PNWNAmet (for North America)^10^, NRCANmet and the Adjusted and Homogenized Canadian Climate Data (AHCCD, for Canads)^11^ and EOBS (for Europe)^12^. Although some are available for shorter time periods, various gridded regional products have proven their importance for in depth climate change and variability analyses. Such examples include HISTALP (for the Greater Alpine Region)^13^, CARPATCLIM (for the Carpathian Region)^14^, ROCADA (for Romania)^15^. Such datasets provide high-resolution climate information tailored for specific geographic regions, enabling studies of regional climate patterns and trends. Studies addressing long-term climate variability also address other regions, such as Asia^16,17^ or Africa^18^, but many areas still lack decent continuous meteorological data series^19^. Satellite imagery has become an important and useful provider of climate or ancillary data^20,21^, but it covers discontinuously only the recent few decades and the results are mostly relevant for clear-sky conditions. The ground-based measurements performed within established meteorological networks in agreement with the World Meteorological Organization’s (WMO) recommendations remain a valuable data source for climate research. The study of the mechanisms that control climate variability can provide relevant results only by using accurate data, including a well-documented understanding of the associated metadata. Changes in location, procedures, instruments, or personnel of the weather stations usually bias the natural variability of the climatic fluctuations that occur without any human influence^22^. The extended development of climate applications and services focuses on using improved data^23^, to enhance outcome quality and facilitate user adoption. The provision of high-quality climate data sets for climate analyses is based on processing data in several steps, including network selection, quality control, homogenization, and validation^24^. Homogenization removes outliers, jumps, and artificial trends in station time series due to non-climatic factors that may trigger inconsistencies in climate change analysis^25^. A few homogenization algorithms can be used to improve the homogeneity of climate data, and the selection of the methods depends on factors like the variable to analyse, the user’s experience, or the level of complexity of the technique^26^.

Homogenized data sets have been made available for many countries, and they support long-term analysis, focusing on different variables at various spatial and temporal resolutions. Air temperature (*T2m*) is most commonly treated^27–29^, but other variables have also been considered, such as precipitation (*PREC*)^30,31^, wind^32–35^, or humidity^36,37^.

The interest in homogenizing climate data series in Romania started at the beginning of the 2000s^38^, and homogenized data sets were used for the country-scale analysis of wind^39^, or for the regional study of *T2m* and *PREC*^40^. Homogenized gridded data sets of ten climate variables were made available for the Carpathian region, including about 2/3 of Romania, covering 1961–2010^14^. Romania also has a comprehensive gridded homogenized climate dataset known as ROCADA (ROmanian ClimAtic DAtaset), covering the period 1961–2013^15^. Daily and sub-daily data homogenization for 2009–2017 was performed for *T2m* using four independent meteorological networks covering Romania and its surroundings^41^.

Considering the changes in measurement techniques, station relocations, and other factors that affected the meteorological network in Romania since the beginning of the measurements (i.e., the 19th century), the homogenization of the data sets is constantly required to ensure the consistency of the results.

This study aims to provide the first homogeneous, gap-free, country-wide data set from 1901 to 2023, which can be used for the long-term variability and trends analysis of *T2m* and *PREC* over the territory of Romania. This dataset is crucial for assessing climate variability and change in Romania, and it supports climate and climate-related applications, such as climate modelling, biodiversity or water resources studies. For example, the dataset is valuable for assessing the long-term variability of droughts at a regional level, as well as for understanding the migration of the isotherms under the climate change influence.

### Schematic overview

**Fig. 1:**
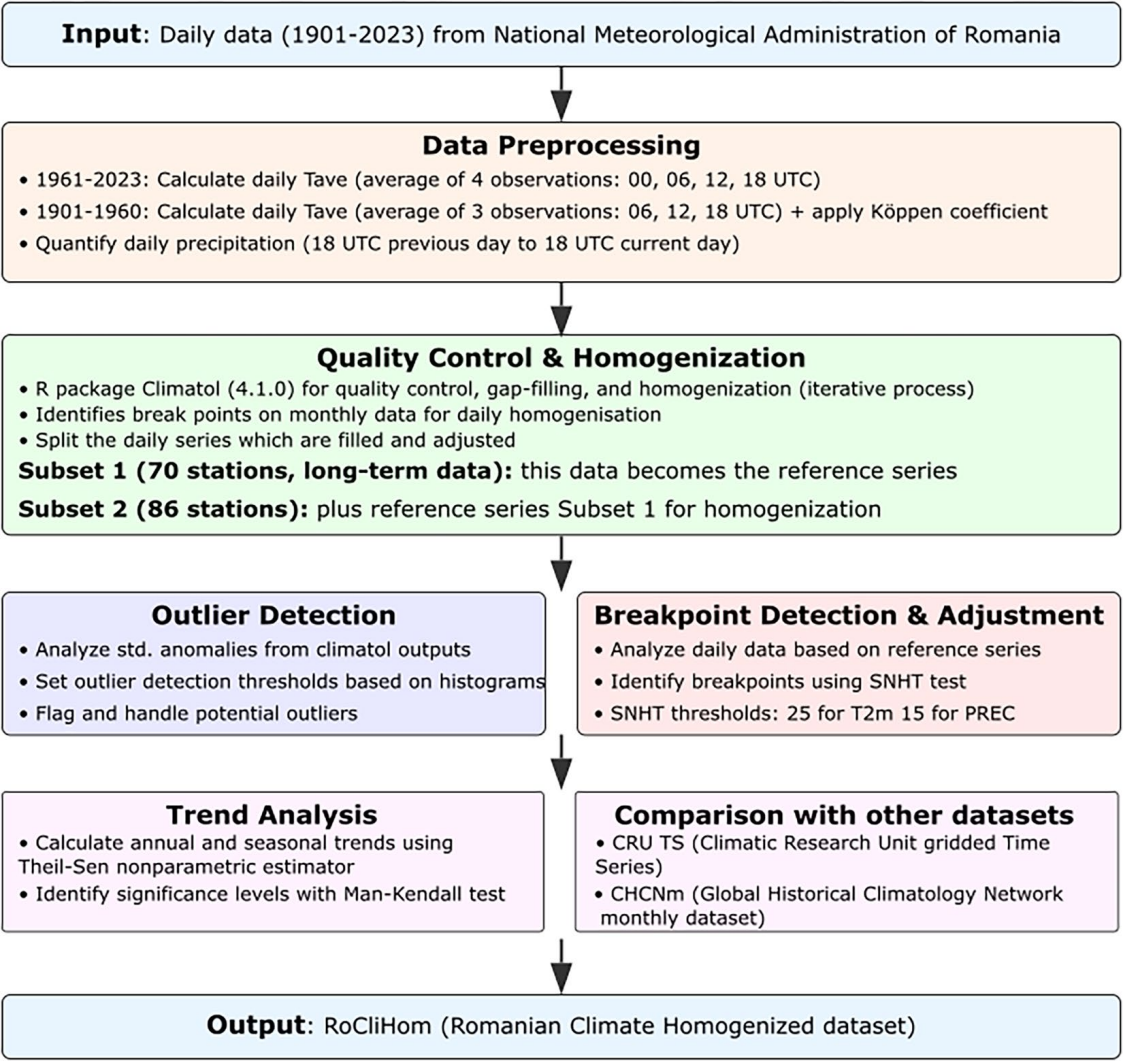
Homogenization procedure and trend analysis workflow.

The RoClimHom homogenization procedure is based on a workflow that includes several stages needed to produce a high-quality, consistent, and robust climate dataset, as follows (Fig. 1):

(i) Data Input: Daily *Tavg*, *Tmax*, *Tmin*, and *PREC* data were collected from 156 weather stations across Romania, spanning the period 1901 to 2023.
(ii) Data Preprocessing: Initial data cleaning and formatting were performed, including qaulity controll, handling missing values, and historical climate data was processed using period-specific methods for temperature averaging and standardized precipitation measurements.
(iii) Quality Control: The quality control process involved identifying and correcting outliers, ensuring that the data adhered to expected ranges and patterns for each variable.
(iv) Homogenization: A key step in the study was the detection and correction of breakpoints—sudden changes in the data caused by non-climatic factors such as station relocations or changes in instrumentation.
(v) Trend Analysis: Once the data were homogenized, long-term trends in *Tavg*, *Tmax*, *Tmin*, and *PREC* were analysed. This step included both spatial and temporal assessments to understand climate variability across Romania.
(vi) Comparison with other datasets: The homogenized data was validated against existing datasets, such as CRU TS (Climatic Research Unit gridded Time Series)^9^ and (ii) Global Historical Climatology Network monthly dataset (GHCNm)^42^.
(vii) Data Output: The final output is a set of homogeneous, gap-free time series for 156 weather stations covering the national territory of Romania (RoCliHom).

### Potential applications

This homogenized dataset, made publicly available, provides a unique resource with significant potential for scientific research and climate-related applications.

By addressing the historical inconsistencies in Romania’s climate data, this study fills a crucial gap in the availability of high-quality, long-term climate records. It provides a robust foundation for future climate research in Romania and beyond, offering insights into both the past and potential future climate dynamics of the region. It can be used to assess historical climate trends, climate scenarios data validation, and evaluate the impact of climate variability on various sectors such as agriculture, water resources, and energy.

Policymakers can leverage the data to develop climate adaptation and mitigation strategies tailored to Romania’s specific climate conditions. Additionally, the dataset could support regional studies, especially in the context of the broader Carpathian region, and contribute to global climate research efforts.

## Methods

### Study area and the historical development of the meteorological network

**Fig. 2:**
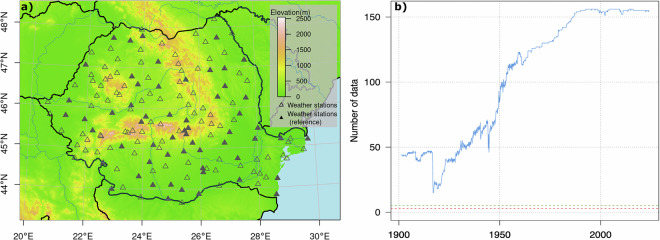
Spatial distribution of weather stations, with the reference network indicated by filled triangles (**a**), and the evolution of the data availability at all stations after 1901 (**b**).

This study encompasses Romania’s national territory. The topographic features of the country are reflected by a wide variety of landscapes and landforms, with altitudes ranging from 0 to 2,544 m.a.s.l., including the Carpathian Mountains, rolling hills, and low plains, with an equal share of 1/3 each (Fig. 2a).

The spatial distribution of the 156 weather stations evenly spans the entire territory of Romania, covering all altitude levels. The 70 stations used as reference stations (i.e., homogenization of the data subset containing the long series) also follows an even spatial distribution of the homogenization process (see the section *Study design and workflows*) (Fig. 2a). Comprehensive metadata with the coordinates and altitude of the stations included in this study are provided in Supplementary information Table S1.

Daily mean (*Tavg*), maximum (*Tmax*), and minimum (*Tmin*) air temperature and precipitation (*PREC*) data available between 1901–2023 measured at 156 weather stations from the Romanian National Meteorological Administration Network, have been selected in the study. This study uses data from weather stations with at least 30 years of available daily measurements, covering the entire territory of Romania. The number of weather stations operating according to the above-mentioned criterion on the Romanian territory over 1901–2023 is presented in Fig. 2b. During this period, the development of the national meteorological network has been shaped by some important biases: (a) two significant declines during the two world wars; (b) a rapid increase of the number of stations until the 1950s; c) implementation of new measurement standards starting 1961, while the network continued to expand steadily until the 1990s; (d) relatively stable number of stations from 1990 to day; e) introduction of automatic measurements after 2001. A brief history of the development of the meteorological network over the study area is presented below. Data before 1901 are geographically sparser and less consistent, and they will be analysed in a further study.

On the current territory of Romania, the activity of meteorological measurements and observations started before the official establishment of the Romanian Meteorological Institute in 1884. For example, meteorological stations operated in Iasi (1770), Bucharest (1773), Cluj (1833), Alba Iulia, Sibiu (1843), Sulina, Oraviţa, Braşov, Baia Mare, Satu Mare (1857), Craiova (1881), Târgu Mureș, Arad, and Petrosani (1883). Some of these early records are no longer available today, but the observation points are still in the national surface network, even if their position has changed over time in an area of several square kilometers. Other observational points were established in the late 1800s and early 1900s, but they ceased their activity around 1960.

In other cases, even if some of these observational points were established as early as 1886 (Cumpana - Arges county), 1890 (Armasesti - Ialomita county), 1892 (Heresti - Ilfov county), 1895 (Casimcea - Tulcea county), 1896 (Strehaia - Mehedinti county), 1897 (Draguseni - Covurlui/Galati county), 1898 (Isaccea - Tulcea county, Studina - Romanati/Olt county), 1899 (Govora - Vâlcea county, Babadag - Tulcea county, Ghimpati - Vlasca/Giurgiu county), 1900 (Gaesti - Dambovita county), 1901 (Retivoiu - Prahova county, Faget - Prahova county, Rucar - Arges county), 1903 (Scropoasa - Dambovita county), 1904 (Perieti - Ialomita county), they did not function continuously, presenting large gaps (e.g.: Strehaia 1896–1916, 1927–1960; Studina 1898–1911, 1944–1960; Govora 1899–1944, 1947–1953, 1955–1958). The majority of these stations ended their activity around the year 1960.

Due to the start of economic development, the public interest in meteorological information grew steadily and led to the establishment of the Romanian Meteorological Institute in 1884. Initially, the institute had as its main priorities the expansion of the surface observational network and the purchase of meteorological professional equipment. The development of the meteorological network was hindered by World Wars I and II, along with the economic crisis, leading to a slow increase in the number of stations and the discontinuation of meteorological activity in some areas.

In 1960, Romania’s surface network comprised 102 weather stations with synoptic programs and 230 weather stations with climatological programs, while the meteorological instruments and the methodologies used for data processing were not fully homogeneous. From 1961, all the stations adopted consistent meteorological manuals for observations and measurements and professional instruments, according to the technological development of the time. Between 2001 and 2015, all weather stations were equipped with automatic instruments at least for measuring air temperature, air humidity, atmospheric pressure, wind direction and speed, and precipitation.

### Data preprocessing

The primary input consists of daily data extracted from the database of the National Meteorological Administration of Romania. For 1901–1960, the daily *Tavg* was derived as the average of three climatological observations taken at 06, 12, and 18 UTC, then a Köppen coefficient was applied, calculated based on the *Tmin* and month of the year. For 1961–2023, the daily *Tavg* was calculated as the arithmetic average of four climatological observations recorded at 00, 06, 12, and 18 UTC.

For the entire period of analysis, daily *PREC* was the total accumulation from 18 UTC on the previous day to 18 UTC on the current day, with the timestamp corresponding to the end of the 24-hour accumulation period. Although the daily database has undergone rigorous quality control and validation procedures, as documented in previous studies^15,41,43,44^, validation is also performed before the homogenization, using the methodology implemented in *Climatol*.

The climatological database is subject to continuous quality control (QC) and validation: in near real-time for newly acquired data, and periodically for historical archives using various methods.

For near real-time QC of air temperature variables (T2m, Tmax, Tmin), values are checked against predefined limits, daily records, and neighboring stations. If any input data used in their calculation is flagged as suspect, the derived temperature values (e.g., monthly values) are also marked as suspect. Precipitation (PREC) validation ensures consistency between recorded amounts and precipitation type (e.g., PREC > 0 cannot coincide with “no precipitation” flags, and vice versa). It also verifies that daily PREC is not exceeded by shorter-interval accumulated measurements (e.g., 1 h, 3 h, 6 h, or 12 h accumulations) from the preceding 24 hours. Violations of these criteria, such as daily PREC being less than accumulated sub-daily PREC or mismatched precipitation-type indicators, trigger data quality flags, prompting expert review.

For historical data, a suite of automated QC tests is implemented [41]. These include checks for: climatic limits, temporal persistence, abrupt shifts (stepwise changes), and spatial consistency. These tests utilize historically derived thresholds, maximum acceptable changes, permissible step differences, and cross-validation based on a geostatistical multivariate interpolation method to flag potentially erroneous measurements. Specifically, data are flagged if values exceed climatological bounds, exhibit unrealistically low variability, display abrupt shifts, or deviate significantly from spatially interpolated estimates based on standardized statistical criteria.

The Climatol homogenization function works with normalized values to make observations comparable between different stations despite potential orographic effects. The recommended normalization is to divide precipitation values by the mean of the series and to standardize temperature data (remove the mean and divide by the standard deviation). All values in any series are estimated as the mean of the corresponding values in nearby stations (always in normalized form). The estimated values are used either to fill in the missing data (undoing the normalization) or to calculate spatial anomalies. These anomalies are the basis for quality control by rejecting outliers that exceed a prescribed threshold.

In addition to the regular quality control performed on the climatological database, the Climatol quality-control procedure was also employed. This procedure uses histograms of standardized anomalies for all stations to optimize the thresholds for outlier rejection. As a result, 35 outliers were identified and subsequently checked against the archive of hard-copy tables. Based on expert judgment, only five outliers were confirmed: one for precipitation, two for average temperature, one for maximum temperature, and one for minimum temperature. Although some anomalous data may reflect correct values due to local weather phenomena, we eliminate them before homogenization to prevent unwanted perturbations in neighboring series. These data are later restored into the homogenized series.

### Study design and workflows

Quality control, data filling, and homogenization of the selected datasets utilised the R package *Climatol*, version 4.1.0. This package, which includes series homogenization and derived products, can be downloaded from the CRAN package repository (https://cran.r-project.org/package=climatol). In addition to meteorological records, *Climatol* utilises stations’ metadata such as coordinates and altitude^45^ and can also use reference series data processed from atmospheric reanalysis^32^.

The *Climatol-based* approach has proven to be effective in the homogenization of air temperature data^28,46,47^, solar radiation^48^, wind speed^33^, wind gusts^32,49^, and precipitation^47,50^. The approach has also been tested against benchmark datasets and has returned results that are comparable to the best other homogenization methods^51,52^. The method normalises the time series and identifies significant shifts (breakpoints) by running the standard normal homogeneity test (SNHT) on the differences between the observed data and the computed reference series. Overall, these studies demonstrate that *Climatol* is a valuable tool for performing homogenization of meteorological data and that the adjustments made by the software do not have a significant impact on the multiannual trends in the data.

Since the majority of the stations were established after the 1950s, we employed a two-step homogenization procedure:

(i) Step 1: homogenization of the data subset including 70 stations, and (ii) Step 2: homogenization of all stations’ data, using as reference the homogenized data obtained after Step 1.

First, a subset of 70 stations with long-term data availability covering the period from 1901 to 2023 was selected. This subset underwent quality control, gap-filling, and homogenization. Further, the long-term homogenized subset served as a reference series for the remaining 86 stations, which had limited data availability for the earlier part of the study period (1901–1960). A similar approach was used by Izsák *et al*.^53^. As shown in previous studies^41,47^, *Climatol* was initially applied to monthly data for each subset to enhance the homogenization results and identify breakpoints. Subsequently, at each station, the daily data were divided into homogeneous sub-periods based on the monthly breakpoints identified in the first stage, which were then filled and adjusted accordingly.

Based on the analysis of different homogenization results and SNHT histograms provided by *Climatol*, SNHT = 25 thresholds were used as a maximum for temperatures, as recommended by Guijarro (2024)^54^, and a threshold of SNHT = 15 for precipitation^47,51^.

The SNHT = 15, 25 thresholds are conservative values (for precipitation and temperature variables), trying to detect and correct most of the significant biases and avoiding false detections, at the cost of letting the smaller biases pass undetected. The monthly homogenizations were repeated with decreasing thresholds of SNHT = 22, 19, and 16, resulting in a substantial increase in the number of detected breakpoints. However, as not knowing the true solution made it impossible to ascertain how many of them were false positives, the authors decided to keep the results obtained with the initial conservative threshold instead of breaking the series into a potentially excessive number of fragments.

To set the thresholds used for the outliers’ detection, we followed the procedure recommended by Guijaro^54^, based on analysing the histogram of standardised anomalies from the *Climatol* runs in exploratory mode.

For the technical validation of the homogenized dataset created, annual and seasonal trends were evaluated using the Theil–Sen nonparametric estimator, and the levels of significance were assessed with the Mann-Kendall test, as implemented in the EnvStats R package^55^.

## Data Records

The dataset is available at the Zenodo repository^56^ with the following access link https://zenodo.org/records/14880417.

The dataset is distributed in long-format CSV files, comprising roclihom_meta_1901_2023.csv for metadata and roclihom_data_1901_2023.csv for observational records.

Roclihom_data_1901_2023.csvt is structured in a way that each row contains the relevant variables at —*tavg* (average air temperature- °C), *tmax* (maximum air temperature - °C), *tmin* (minimum air temperature - °C), and *prec* (precipitation - mm)—along with corresponding flag columns. These flags indicate the status of the data as follows: *0* for original values, *1* for only gap-filled data, and *2* for corrected (homogenized) values. Additionally, the dataset includes a station identifier (id), as well as temporal information (Table 1).

**Table 1:** Sample of the dataset for station Bucuresti Baneasa as exrtacted from roclihom_data_1901_2023.csv file.

id	year	month	variable	value	flag
425606	2023	12	prec	27.2	0
425606	2023	12	tavg	4.3	0
425606	2023	12	tmax	8.6	0
425606	2023	12	tmin	0.7	0

The metadata file roclihom_meta_1901_2023.csv, contains details for each station, including station ids, geographical coordinates (in decimal degrees for both longitude and latitude), altitude (in meters), and station names.

## Technical Validation

In this section, the technical quality of the new datasets is validated by assessing the impact of the homogenization procedure. This assessment involves analysing identified breakpoints, comparing trends derived from gap-filled only and homogenized data, and evaluating the influence of potential artificial jumps on the results. Additionally, the newly generated dataset is compared with previously published, similar climate data to further establish its reliability.

### Breakpoints detection

**Fig. 3:**
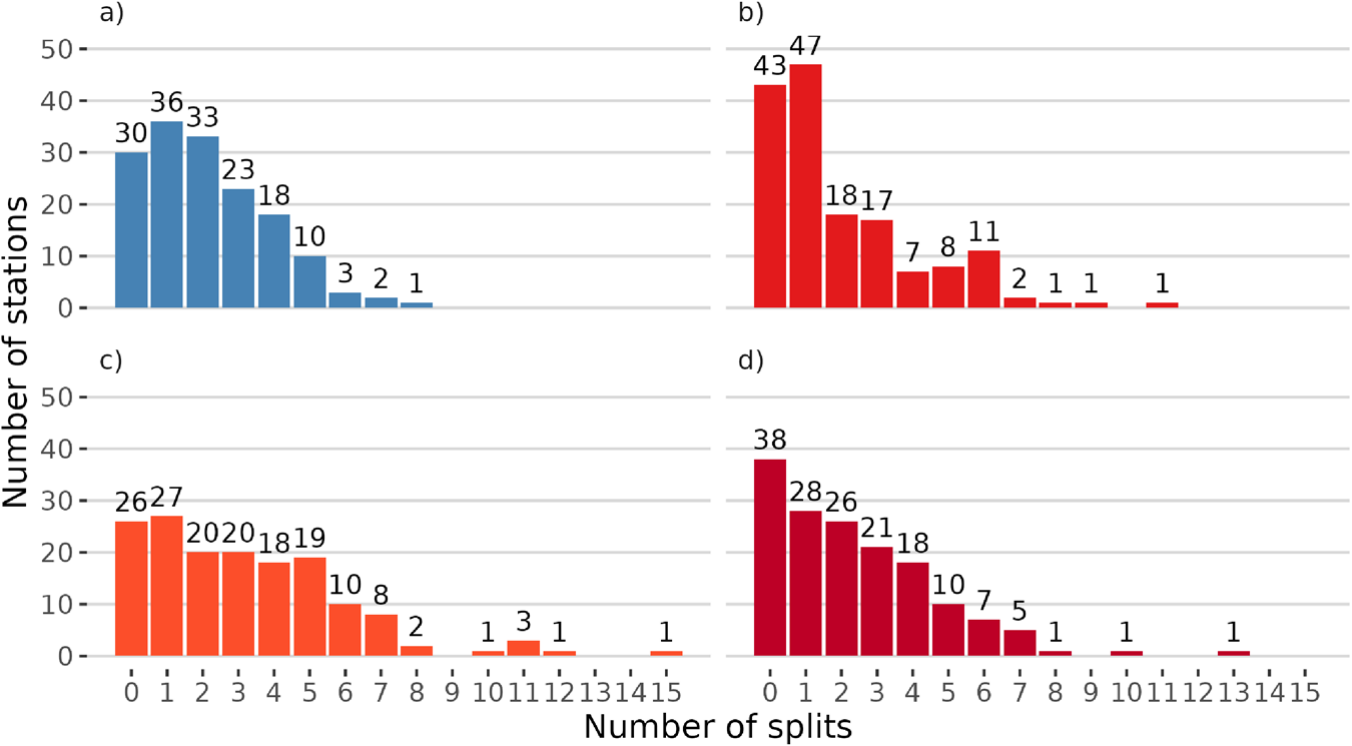
Distribution of stations based on detected breakpoints in the *PREC* (**a**), *Tavg* (**b**), *Tmin* (**c**), and *Tmax* (**d**) time series.

The number of breakpoints for each variable resulting from applying the homogenization approaches is depicted in Fig. 3. The numbers of homogeneous time series are also shown on the graphs. In total, 1,512 breakpoints were detected across all variables, including 333 for *PREC*, 310 for *Tavg*, 496 for *Tmin*, and 373 for *Tmax*. Supplementary information Table S1 also presents the number of breakpoints detected by *Climatol* for each station and variable. The homogenization underscores the prevailing low-count breakpoints across all examined climatic variables, particularly for *Tavg*, where a single break is most commonly observed. The findings suggest that breakpoints are widely detected across the network, but higher counts of such events are relatively rare, especially for maximum and minimum temperatures. The broader range of break detections in *Tmin* highlights it as a parameter with potentially more complex temporal variability in the observed dataset than *Tmax*. For example, the 15 breakpoints detected in *Tmin* at one station (Craiova) are plausible, considering that, upon checking the metadata, the station was relocated 13 times, and the instrumentation was changed several times during the period analysed in this work (1901–2023).

**Fig. 4:**
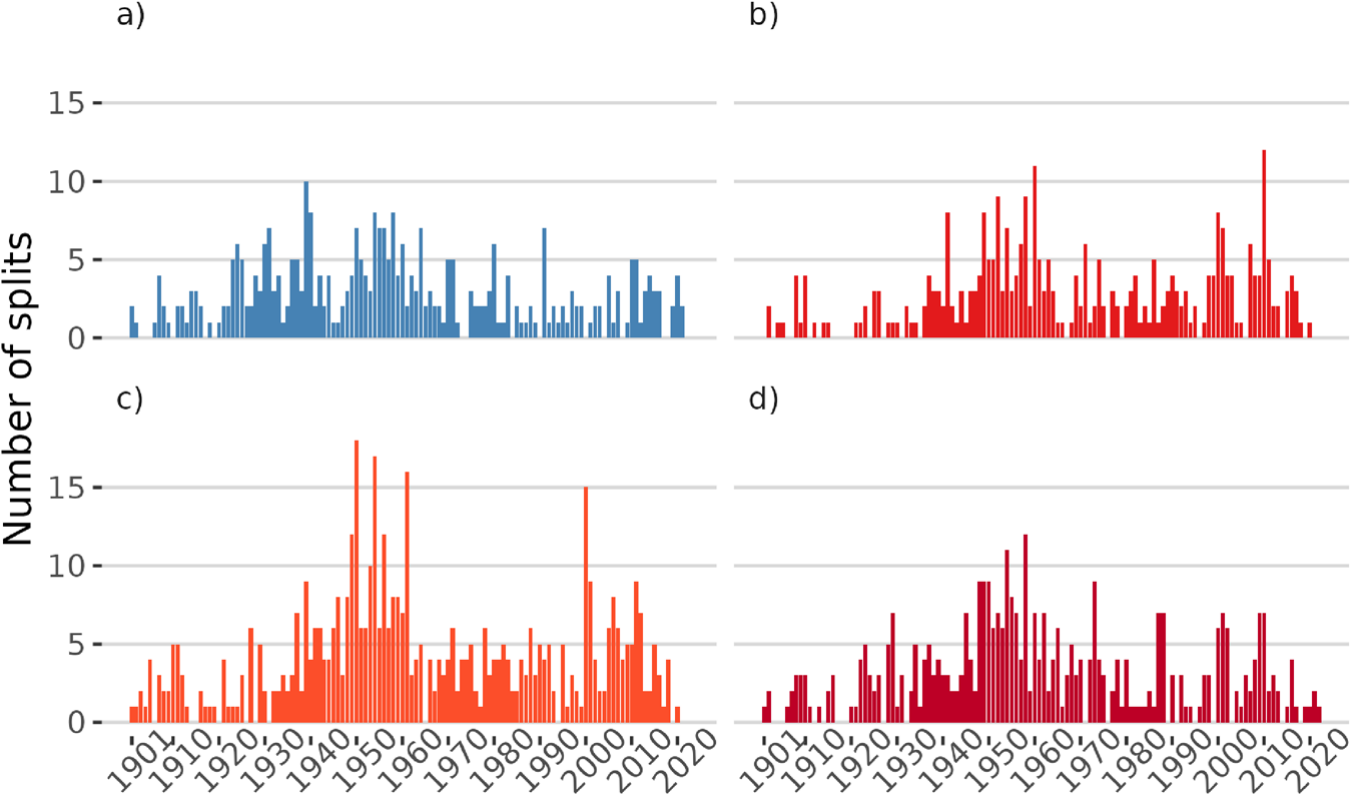
Frequency of detected breakpoints along time in the *PREC* (**a**), *Tavg* (**b**), *Tmin* (**c**), and *Tmax* (**d**) time-series.

Figure 4 details the number of breakpoints detected across the four climate variables analysed between 1901 and 2023. The distribution of breakpoints by year and variable reveals significant anthropogenic influences across different periods, particularly in the mid-20th century, with marked spikes. The concentrated activity around specific periods suggests that these years have experienced notable non-climatic events or transitions, which could be an area for further investigation or cross-referencing with historical climate records. Overall, *Tmin* appears to be the most unstable variable, often showing a higher number of breakpoints, while *PREC* and *Tmax* also spike distinctly in certain years. For instance, certain years—such as 1939, 1959, and 2000—exhibit a particularly high number of breakpoints across all variables, indicating significant observation biases during these periods. These periods may be related to significant historical events or technological advancements, such as WWII, the implementation of new measurement standards in the early 1960s, or the introduction of automatic weather stations at the beginning of the 2000s.

### Trend analysis

Following the development of the RoClimHom dataset, a trend analysis was performed to evaluate the annual and seasonal trends present in air temperature and precipitation parameters. This evaluation was conducted using the non-parametric Mann-Kendall test and the Theil-Sen slope estimator.

First, the trends were computed using two types of datasets: a *Climatol*-only gap-filled dataset and a *Climatol*-homogenized dataset. The purpose of this comparison was to assess the impact of homogenization on the detected trends by comparing the slopes derived from only the gap-filled data with those derived from the homogenized dataset.

**Fig. 5:**
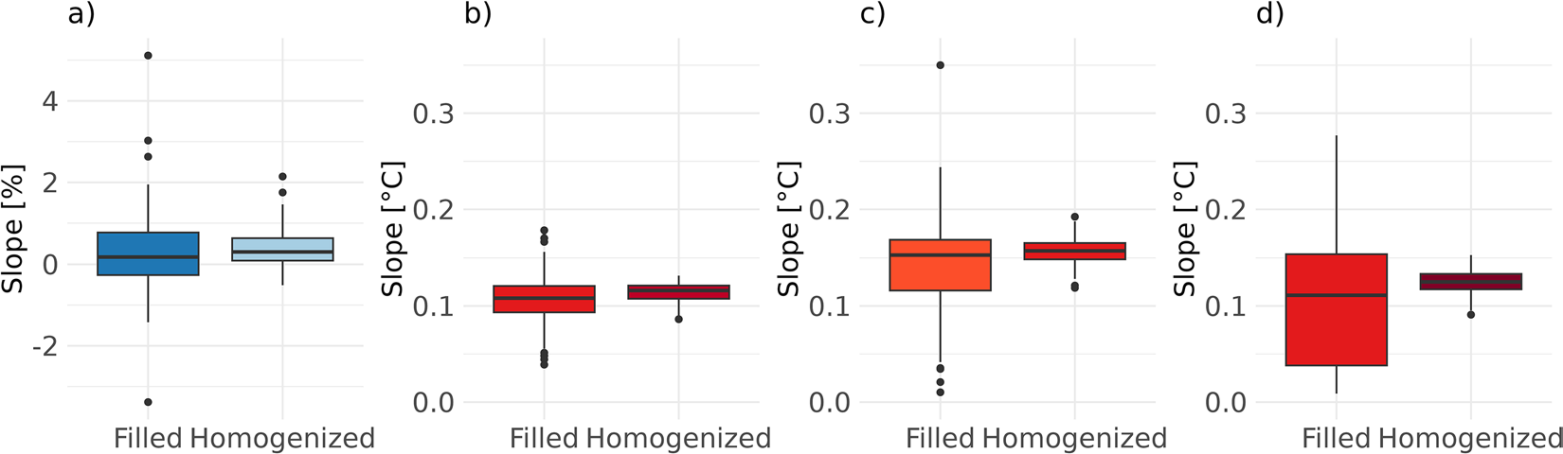
Boxplots of the Theil–Sen’s slope computed for the gap-filled (first boxplot) and homogenized (second boxplot) yearly time series of *PREC* (**a**), *Tavg* (**b**), *Tmin* (**c**), and *Tmax* (**d**).

Figure 5 reveals that the homogenization has reduced the magnitude and variability of trends, particularly for *PREC* and *Tmax*, which demonstrates that the homogenization methods effectively remove biases in the data. Gap-filling, on the other hand, might introduce bias due to the way missing data is filled. There is no evident change signal for *PREC*, although positive trends were computed for most stations (130 out of 156). The trends in *T2m* are positive in all variables (indicating warming), with some variations, which is consistent with other climate change studies^57^. Gap-filled data generally show a greater spread in the slope values, with wider interquartile ranges and more outliers. Homogenized data have more consistent and less variable slopes, indicating a more stable trend than the raw, inhomogeneous data.

**Fig. 6:**
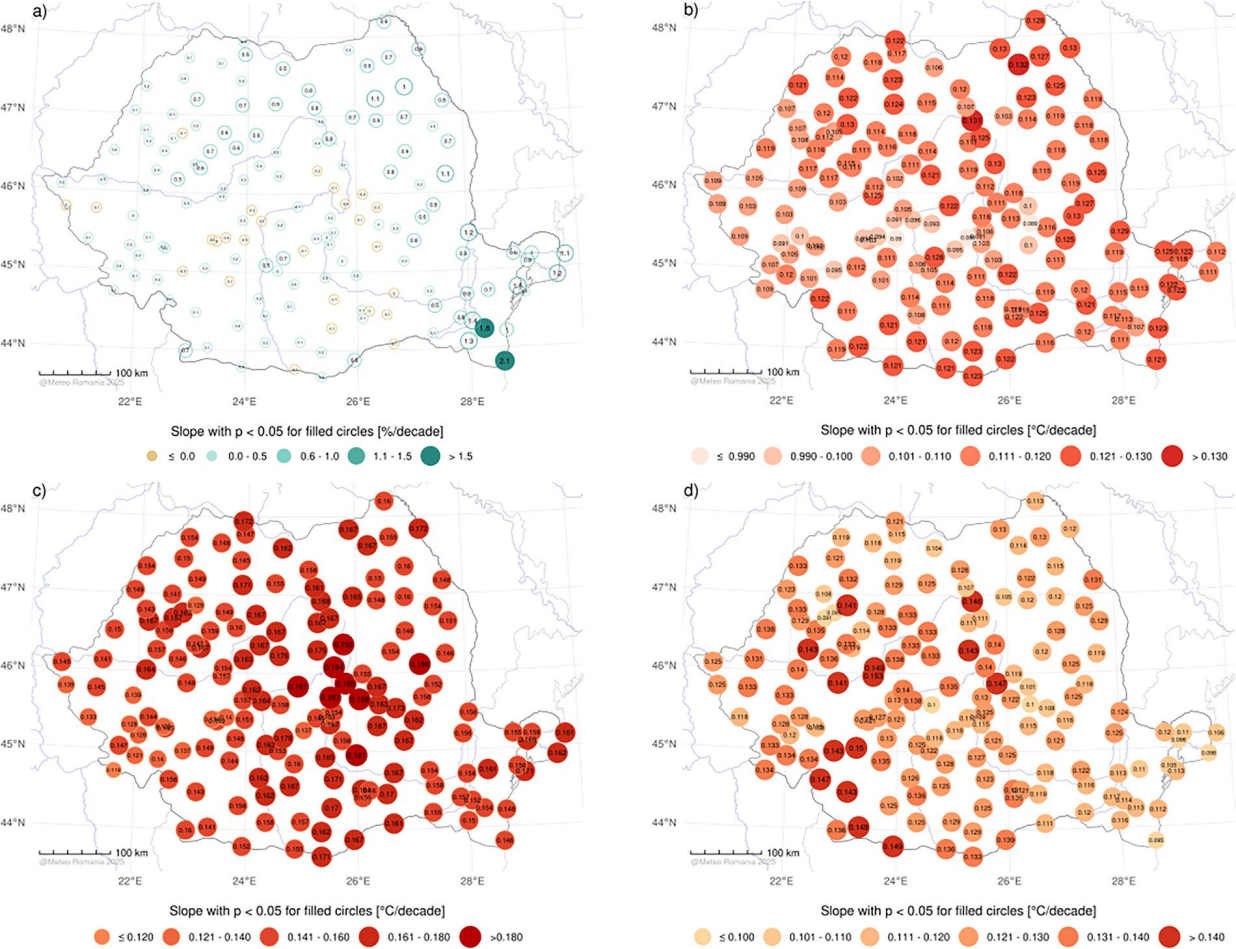
Annual Theil–Sen’s slopes and Mann-Kendall significance levels (filled circles) evaluated on homogenised yearly time series of *PREC* (**a**), *Tavg* (**b**), *Tmin* (**c**), and *Tmax* (**d**) time-series.

Spatial distribution maps of the annual Theil–Sen’s slopes and Mann-Kendall significance levels (p < 0.05 filled circles) evaluated for homogenized *PREC* (%/decade), *Tavg*, *Tmin*, and *Tmax* (°C/decade) - confirm the previous trend analysis (Fig. 6). Positive trends in *PREC* were observed at most stations, but their magnitudes are generally weak, with statistically significant at only  two locations. Conversely, the strong positive trends in *T2m* are statistically significant across all stations and for all three temperature variables.

The *PREC* trends analysis reveals a predominance of positive trends, with approximately 83.3% of the analysed stations (130 out of 156) showing increasing slopes. This distribution suggests a general trend of increasing precipitation amounts at the regional level (Fig. 6a). The analysis of temperature trends across 156 stations reveals a consistent and statistically significant increase in *Tavg*, *Tmin*, and *Tmax (*Fig. 6b–d). Overall, the *Tavg*, *Tmin*, and *Tmax* trends strongly suggest that the observed increases are part of a broader pattern of climate change, consistent with other results at the continental level^57^. In this context, the data sets can be extremely useful in regional applications focusing on water deficit and excess, heatwaves or territorial planning.

Table 2 summarises the statistics of increasing and decreasing long-term trends for the four climate variables analysed in this study, highlighting both statistically significant (p-values < 0.05) and overall trends. *PREC* trends display more variation, with significant positive trends predominantly observed during winter (DJF) and summer (JJA), while autumn (SON) reveals a notable balance between positive and negative trends. In contrast, the trend statistics indicate a consistent increase in *Tavg*, *Tmin*, and *Tmax* across all seasons, with almost all trends exhibiting positive changes and no significant negative trends detected.

**Table 2:** Percentage of stations revealing positive (+) and negative (−) trends in *PREC*, *Tavg*, *Tmin*, and *Tmax* (1901–2023).

Variable	Time interval	S* (+)	S* (−)	(+)	(−)
*PREC*	Annual	1.28	0	83.3	16.7
	DJF	7.69	4.49	75.0	24.4
	JJA	9.62	1.92	64.7	35.3
	MAM	1.28	1.28	69.9	29.5
	SON	0.641	1.28	37.2	62.8
*Tavg*	Annual	100	0	100	0
	DJF	92.9	0	100	0
	JJA	99.4	0	100	0
	MAM	93.6	0	100	0
	SON	46.8	0	100	0
*Tmin*	Annual	100	0	100	0
	DJF	100	0	100	0
	JJA	98.7	0	100	0
	MAM	100	0	100	0
	SON	99.4	0	100	0
*Tmax*	Annual	100	0	100	0
	DJF	98.7	0	100	0
	JJA	90.4	0	100	0
	MAM	82.7	0	95.5	2.56
	SON	50	0	99.4	0

S* stands for significant trends (p-values < 0.05). The ‘no trend’ cases are not shown in this table.

**Fig. 7:**
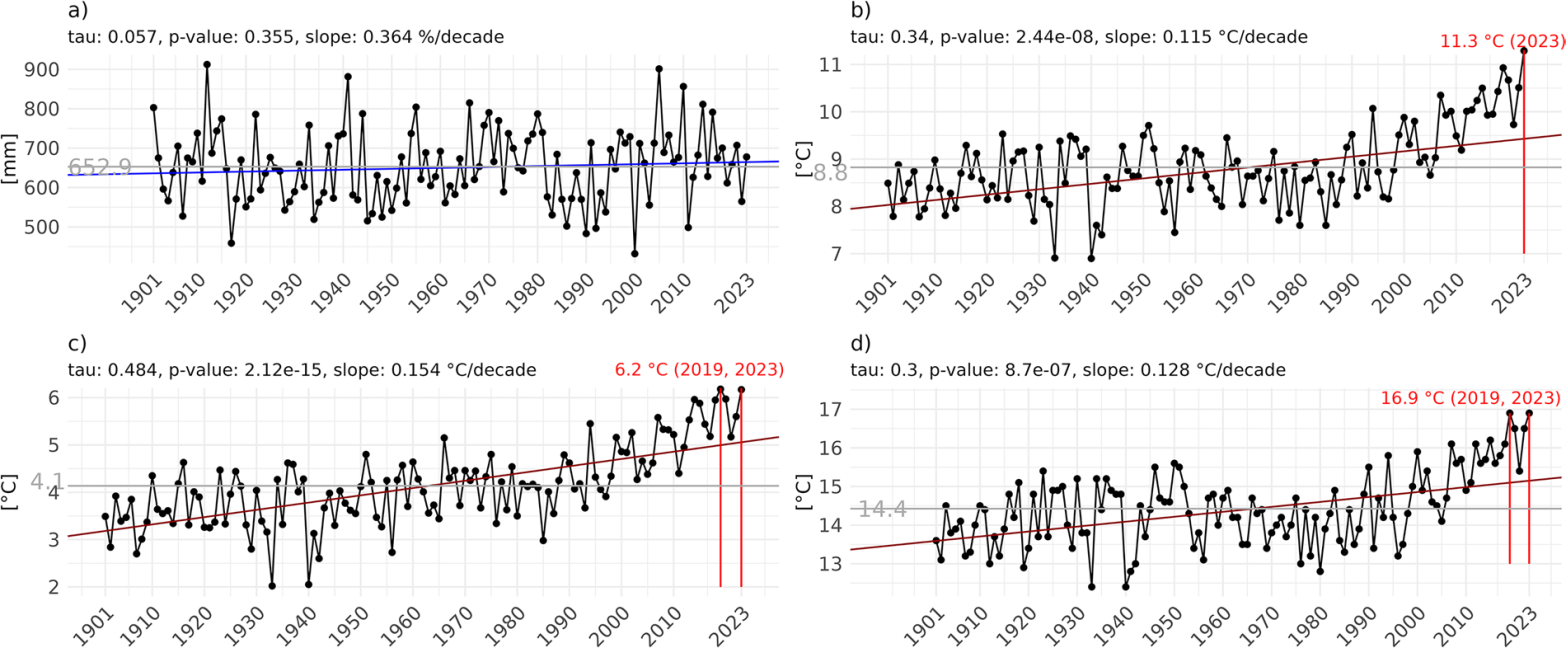
Trends at the country level for *PREC* (**a**), *Tavg* (**b**), *Tmin* (**c**), and *Tmax* (**d**), computed from the homogenized dataset. All graphs include statistical information such as Mann-Kendall tau, p-value, and Theil-Sen’s slopes, which provide quantitative measures of the trends’ significance and magnitude. Vertical red lines indicate when record-breaking air temperatures occurred, specifically in 2019 and 2023. The plots show the multiannual mean (1901–2023) for each variable as dark grey lines.

The country-scale average time series plots reveal no significant change in *PREC*, while *Tavg*, *Tmax*, and *Tmin* exhibit statistically significant increasing trends, indicating a general warming over the 20th century and the first decades of the 21st century (Fig. 7).

For *PREC*, the data points representing levels over time show a generally stable trend around 650 mm, with little to no slope, closely matching the multiannual mean of 652.9 mm. The p-value (0.355) suggests that the observed trend is not statistically significant, implying that there have been no significant changes in *PREC* over the given period (Fig. 7a). *Tavg* has increased over time, with the trend line showing a consistent upward trajectory. The p-value (2.44e-08) indicates that the observed upward trend in Tavg is highly statistically significant, with a slope of 0.115 °C/decade (Fig. 7b). *Tmin* also exhibits an upward trend, with the red trend line showing a clear increase. The p-value is highly significant (2.12e-15), suggesting a robust upward trend in Tmin of 0.154 °C/decade (Fig. 7c). The *Tmax* trend is similar to *Tavg*, namely less steep than the *Tmin* (0.128 °C/decade) (Fig. 7d).

It is worth noting that 2023 was characterised by exceptional climate events, such as the record-breaking maximum value of *Tavg* (11.3 °C at the country scale), or matching the record high values for *Tmin* (previous highest value 6.2 °C in 2019) and *Tmax* (previous highest value 16.9 °C in 2019).

Spatial distribution and time series trend plots for the seasonal subsets for each variable are shown in Supplementary Information (Figs. S1–S8).

### Case study

**Fig. 8:**
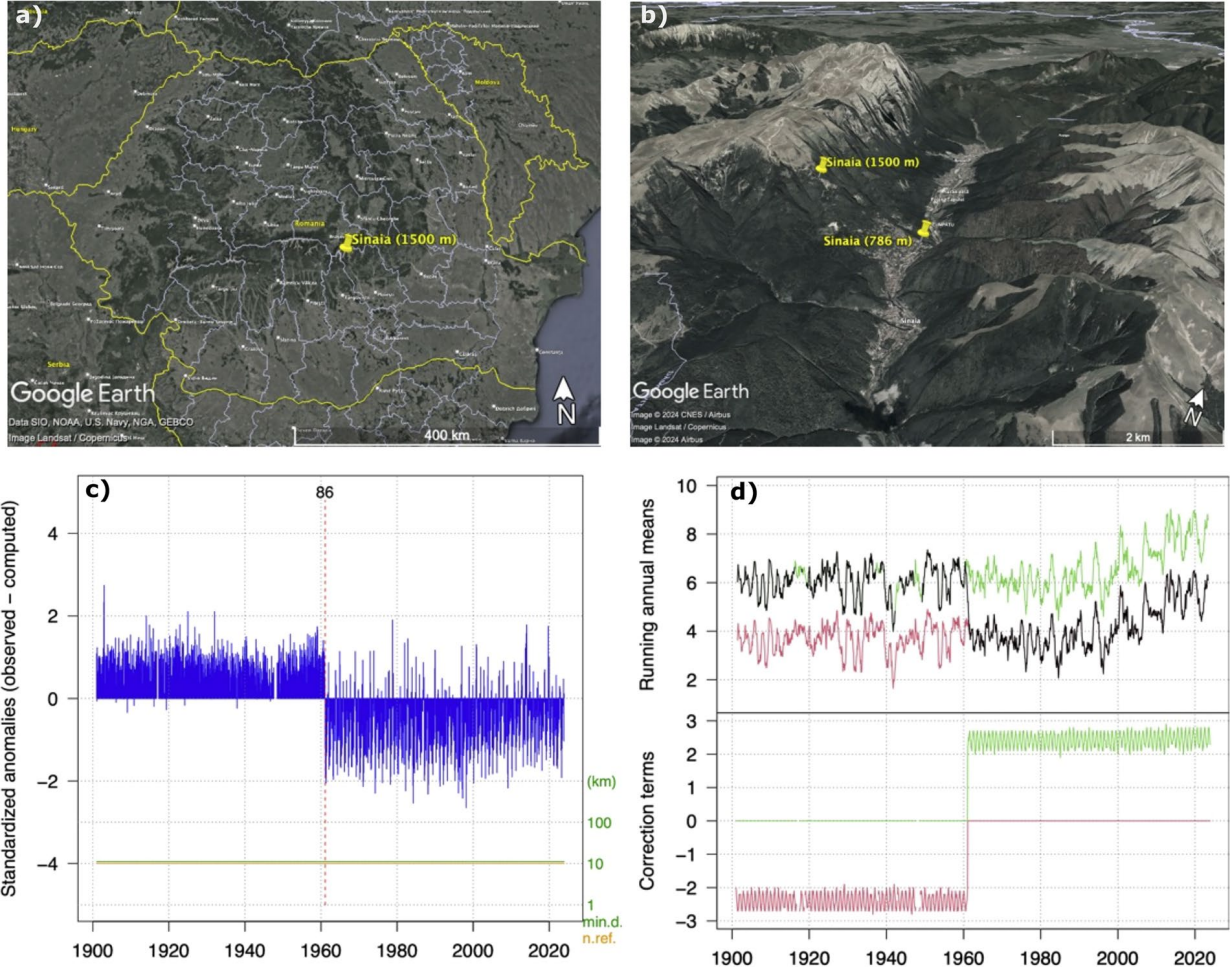
The location of the Sinaia weather station in Romania is shown in the upper left (**a**). The current location of the Sinaia station (1500 m a.s.l.) and its former location (1886–1960 at 786 m.a.s.l.) are shown in the upper right (**b**). In the lower left is shown the example of breakpoint detection at the Sinaia 1500 station, in the monthly average air temperature (**c**), and the lower right (**d**) depicted the two-time series reconstructions, for both homogeneous sub-periods (running annual means on Y-axis, shown by the green and red lines) along with the applied corrections (correction terms on Y-axis, relative to the original records shown by the black line) (plots extracted from the *Climatol* output).

The advantages of using homogenized data sets for improving the consistency of the data by employing homogenization are explored using the Sinaia 1500 weather station case, where the site relocation biased the meteorological time series. The case study is also relevant for snow-related studies, as it shows the sensitivity of the data to site location. Metadata analysis indicates that the station was relocated at the end of 1960 from Sinaia city, situated at approximately 786 m a.s.l., to a nearby location at a higher altitude, i.e., 1500 m a.s.l. (Fig. 8a,b). The raw data analysis highlighted a major shift in both air temperature (Tavg, Tmin and Tmax) and precipitation time series during the 60 s. For exemplification, Fig. 8c,d illustrate the inhomogeneities induced by site relocation in the *Tavg* time series.

This shift is identified in the running annual mean series of air temperature in February 1961 (SNHT value 86). The two lines (green and orange) at the bottom of the breakpoint detection graphs represent the minimum distance between neighbouring data points (green) and the number of reference data points (orange), with a logarithmic scale applied to the right axis for these additional lines (Fig. 8c). The annual running mean of the reconstructed time series is depicted in the plot on the right side of the panel (Fig. 8d). Corrections applied to each time series are colour-coded at the bottom of the corresponding graph, while a black line represents the original time series. This relocation was detected not only in the *Tavg* time series but also in the *Tmin* and *Tmax* series (both in February 1961), as well as in the *PREC* time series (in November 1960). No long-term climatological analysis could be conducted on the unhomogenized time series for this station. Notably, the computed Theil-Sen trend of the gap-filled *Tavg* data for the period 1901–2023 was negative, whereas the trend for homogenized data is positive, i.e., 0.103 °C/decade.

### Comparison with other datasets

The homogenized *Tavg*, *Tmin*, *Tmax*, and *PREC* datasets for 1961–2023 produced over the weather stations in Romania were evaluated against existing datasets commonly employed in long-term climate analysis, specifically (i) CRU TS^9^ and (ii) GHCNm^42^. Comparing our dataset with CRU TS and GHCNm provides a broader perspective on how different homogenization and optimization approaches influence the final outputs, offering insights into the reliability and consistency of our dataset in a global context. Although all three datasets ultimately originate from the same ground-based measurements (at least partially), the differences in homogenization and optimization procedures make them valuable benchmarks for validating our newly created dataset. Each dataset employs distinct methods for postprocessing, and evaluating our dataset against theirs provides an opportunity to assess the robustness of our approach.

### CRU TS

**Fig. 9:**
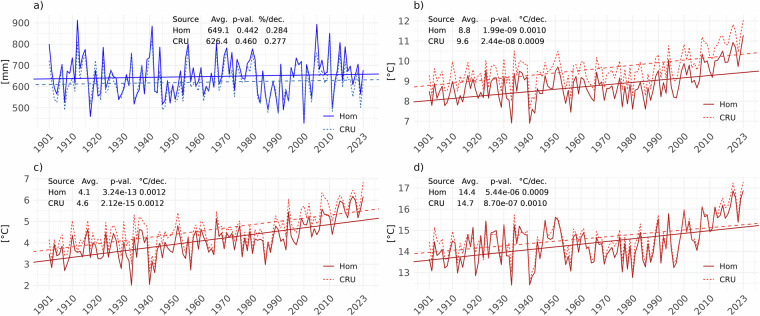
Trends at the country level for both the homogenized (Hom) and gridded CRU TS datasets (CRU), for *PREC* (**a**), *Tavg* (**b**), *Tmin* (**c**), and *Tmax* (**d**).

CRU TS is a global gridded dataset with a 0.5° latitude by 0.5° longitude resolution, covering all land domains of the world except Antarctica. It is generated by interpolating monthly climate anomalies derived from weather station measurements. Figure 9 presents temporal trend plots for the country-averaged variables analysed for both the homogenized and CRU TS datasets. *PREC* data show no statistically significant trend in either dataset, while *T2m* exhibits a strong, statistically significant warming trend for both datasets, very well aligned on the rate of change per decade. However, CRU TS values are slightly higher on multiannual means, indicating a greater warming pattern over the last century. The consistency observed in trends and statistical metrics between the homogenized weather station-based data and CRU TS dataset indicates a high level of agreement between the two datasets, specifically for *Tmax* and *PREC*. Differences in absolute values, which are more noticeable for *Tavg* and *Tmin*, could be explained by the fact that the gridded dataset uses data from a limited number of stations in the Romanian territory, i.e., 40. However, the similarity in trends - particularly the highly significant warming trends in *T2m* - suggests that both datasets capture the same underlying climate signal for Romania over the 1901–2023 period.

### GHCNm

The GHCNm dataset offers monthly climate summaries compiled from numerous weather stations worldwide. The dataset was created in the early 1990s, with methodology updates released in 1997, and 2011, and the latest versions in 2018 for air temperature^58^ and 2024 of precipitation^59^. The length of the stations’ time-series varies, with some of the earliest data being from the 18th century. While certain station records are historical and no longer receive updates, many are still active and provide timely updates that are valuable for climate monitoring. The current iteration, GHCNm v4, includes average monthly temperature data and a beta version of monthly precipitation data.

For air temperature, 40 time series from weather stations covering Romanian territory were identified, whereas for precipitation, only 37 time series were identified in the GHCNm dataset. The scatterplots between the non-homogenized (Raw), homogenized, and GHCNm datasets over the stations in Romania were compared, and several accuracy indicators were computed, such as Spearman correlation, mean absolute error (MAE), and relative root mean squared error (RRMSE).

**Fig. 10:**
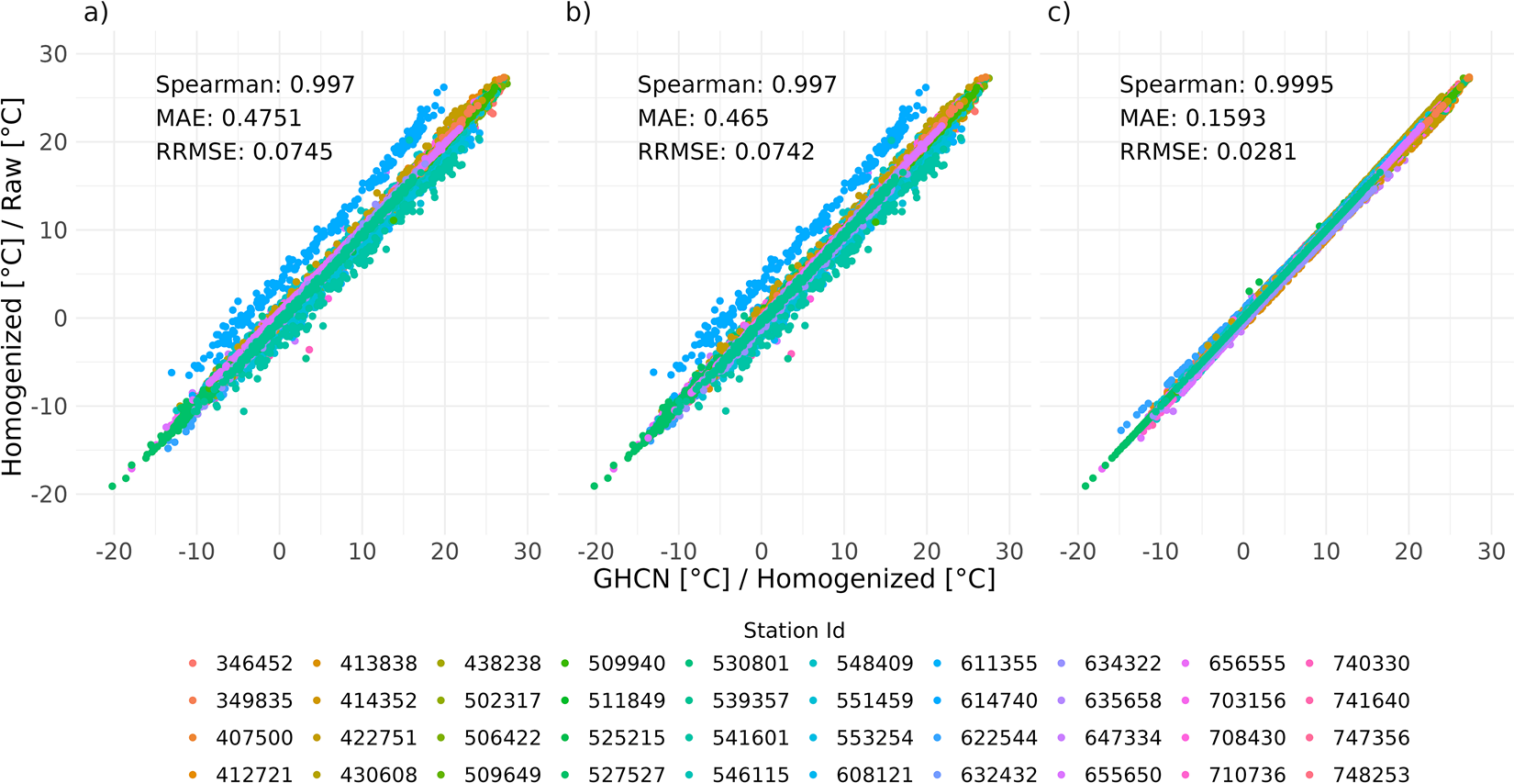
Scatter plots comparing GHCN, homogenized (GHCN vs Homogenized (**a**)) and raw (GHCN vs Raw (**b**)) monthly average air temperatures by station ID. Additionally, the relationship between homogenized and raw data is also displayed (Homogenized vs Raw (**c**)).

The air temperature scatterplots (Fig. 10) illustrate a significant correlation among the GHCNm, homogenized, and raw temperature datasets, with only minor variations observed among the analysed pairs. While there is a strong agreement between the GHCNm and the homogenized datasets, the comparison with the raw data indicates that the homogenized dataset may be more reliable than the GHCNm. The discrepancies between the GHCNm and the Homogenized and Raw datasets can be attributed to the differing methodologies used in computing daily averages. In the homogenized and raw datasets, the average was calculated from synoptic observations taken at 00, 06, 12, and 18 UTC. In contrast, the GHCNm dataset utilized the average between *Tmin* and *Tmax*^60^.

**Fig. 11:**
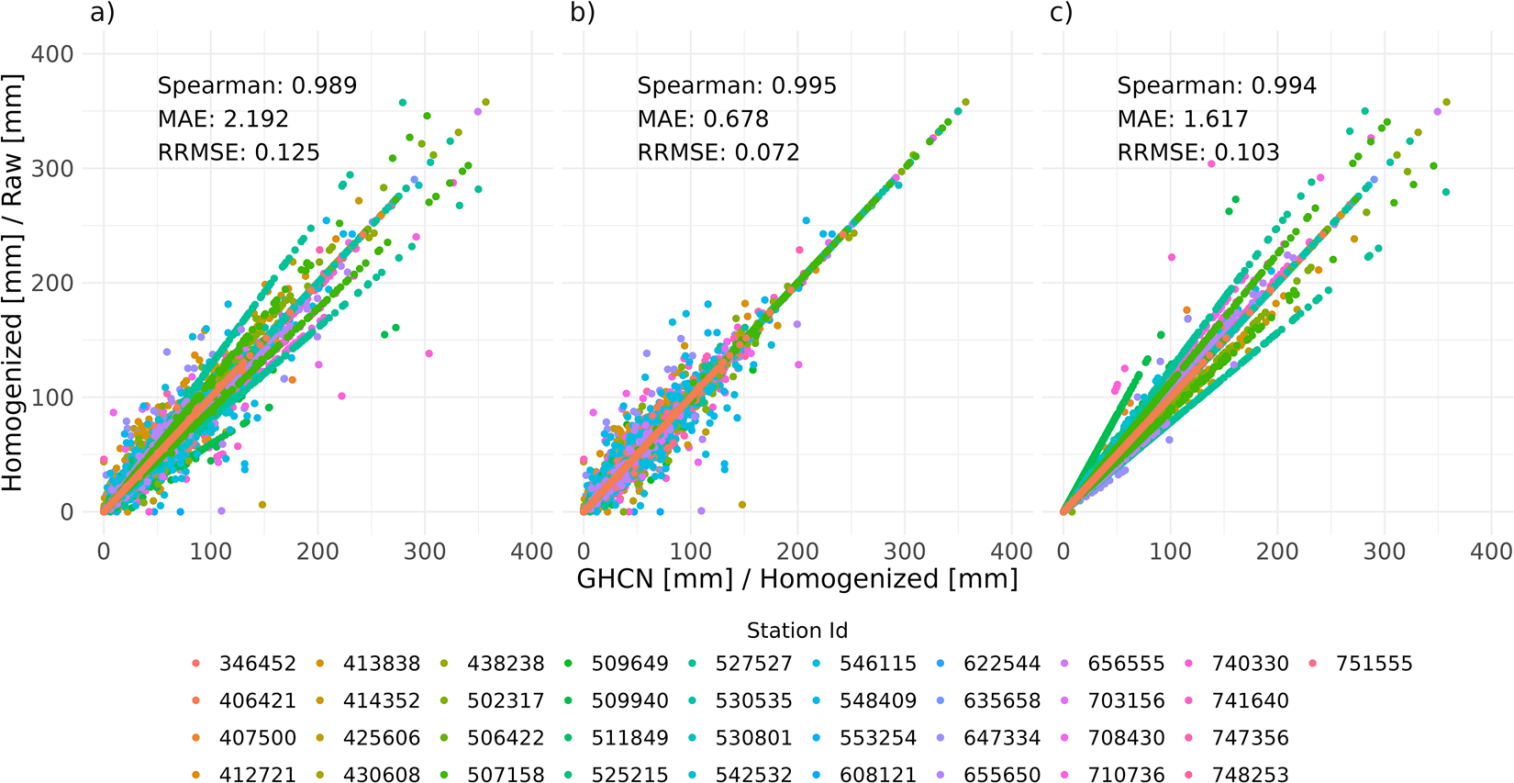
Scatter plots comparing GHCNm, homogenized (GHCNm vs Homogenized (**a**)) and raw (GHCN vs Raw (**b**)) monthly precipitation amounts by station ID. Additionally, the relationship between homogenized and raw data is also displayed (Homogenized vs Raw (**c**)).

The comparison of GHCNm, homogenized, and raw precipitation datasets also reveal strong correlations across all pairings (Spearman’s ≥ 0.989), indicating high consistency between the datasets (Fig. 11). The GHCNm vs. Raw data demonstrates the strongest agreement, which is expected considering that both datasets underwent only quality control, without any homogenization (adjustments) applied to them. Regarding the GHCNm vs. Homogenized dataset, while all comparisons exhibit robust correlations, some discrepancies are observed, particularly at higher precipitation values, which are due to the correction factors applied by the *Climatol* when breakpoints were detected and time series reconstructed.

**Fig. 12:**
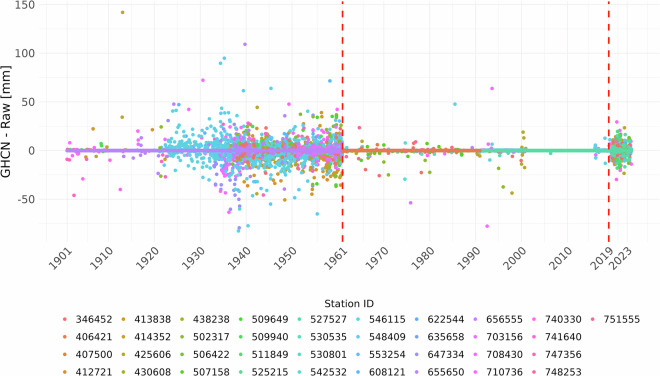
Differences over time (from 1901 to 2023) between GHCN and Raw monthly precipitation amounts by station ID. The years 1961 and 2019, which delineate the periods when the two datasets agree very well, are marked on the plot with red lines.

To better understand the discrepancies unexplained by the corrections applied to the homogenized dataset, the differences between the GHCNm and Raw datasets are shown in Fig. 12. It can be seen that there are two periods with significant differences, both in terms of number and magnitude, namely before 1961 and after 2019, when the data points are highly scattered, with notable deviations from the baseline (0). Even if some differences may be explained by the different reconstruction methods applied to the two datasets, the clustering of the deviations may be attributed to the methodology applied for generating the GHCNm data set. For the Raw dataset, the same measurement unit (mm) and computing method have been used consistently throughout the entire period (i.e., daily precipitation computed as the amount accumulated from 18 UTC to 18 UTC of consecutive days, and the timestamp attributed to the end of the accumulation period). However, for the GHCN data, it cannot be found in the literature how the monthly values were derived or whether other measurement units were involved in the processing of the data.

These findings underscore the overall reliability and consistency of the dataset while highlighting potential areas for further investigation into the sources of differences in the different precipitation datasets.

## Usage Notes

### Important caveats

Undetected Errors: Despite efforts to ensure data quality, undetected errors may still exist within the dataset. Users are advised to remain vigilant for any inconsistencies, particularly when extreme values are observed, and to report these to the authors to improve the dataset.

Data Homogeneity: Although the dataset includes homogenization steps (indicated by flag 2), some inhomogeneities may remain. Biases introduced by factors such as changes in measurement instruments, station relocations, or environmental changes around weather stations may not have been fully corrected.

## Supplementary information

Supplementary information

## Data Availability

No custom code was used in the generation or processing of the datasets. The entire analysis was conducted using functions available in the R^61^ packages climatol^45^, sf^62^, terra^63^, EnvStats^55^, and ggplot2^64^. These packages are distributed under open-source licenses and are publicly accessible via the Comprehensive R Archive Network (CRAN) at https://cran.r-project.org/.
