# Supplementary material for: Long-term homogenized air temperature and precipitation datasets in Romania, 1901–2023
Source: Sci Data. 2025 Jul 1;12:1116. doi: 10.1038/s41597-025-05371-4 (PMC12214662; doi:10.1038/s41597-025-05371-4)
Supplement: Supplementary file 1 — Supplementary information [file 41597_2025_5371_MOESM1_ESM.pdf]

Supplementary information
Long-term homogenized air temperature and precipitation datasets in Romania, 1901 – 2023

Authors
Alexandru Dumitrescu[1], Dana Micu[1], Jose Guijarro[2], Ancuta Manea[1], Sorin Cheval[1,3]

Affiliations
1. National Meteorological Administration, Bucharest, Romania
2. Retired from the State Meteorological Agency (AEMET), Balearic Islands Office, Palma de Mallorca, Spain
3. Doctoral School of Geography, Faculty of Geography, Babeș-Bolyai University, Cluj-Napoca, Romania

corresponding author: Alexandru Dumitrescu (alexandru.dumitrescu@gmail.com)

Table of Contents

Table S1 Stations' metadata and the number of breakpoints detected for each variable and station........ 1
Fig. S1 Seasonal Theil–Sen slopes and Mann-Kendall significance levels (filled circles) evaluated on homogenized yearly time series of precipitation (%/decade)........ 9
Fig. S2 Seasonal Theil–Sen slopes and Mann-Kendall significance levels (filled circles) evaluated on homogenized yearly time series of average air temperature (°C/decade). 10
Fig. S3 Seasonal Theil–Sen slopes and Mann-Kendall significance levels (filled circles) evaluated on homogenized yearly time series of minimum air temperature (°C/decade)... 11
Fig. S4 Seasonal Theil–Sen slopes and Mann-Kendall significance levels (filled circles) evaluated on homogenized yearly time series of maximum air temperature (°C/decade).. 12
Fig. S5 Trends at the country level for seasonal precipitation computed from the homogenized dataset. Mann-Kendall tau, p-value, and Theil-Sen slopes quantitatively measure the trends' significance and magnitude........ 13
Fig. S6. Trends at the country level for seasonal average air temperature computed from the homogenized dataset. Mann-Kendall tau, p-value, and Theil-Sen slopes quantitatively measure the trends' significance and magnitude........ 13
Fig. S8 Trends at the country level for seasonal maximum air temperature computed from the homogenized dataset. Mann-Kendall tau, p-value, and Theil-Sen slopes quantitatively measure the trends' significance and magnitude........ 14

Table S1 Stations' metadata and the number of breakpoints detected for each variable and station

Longitude (°)	Latitude (°)	Altitude (m.a.s.l.)	Station Id	Station Name	Breakpoints detected			
					PREC (mm)	Tavg (°C)	Tmin (°C)	Tmax (°C)
27.9656	44.0883	158	408800	Adamclisi	2	0	0	1
27.1704	46.1047	101	606705	Adjud	0	0	1	0
26.2128	44.5001	90	430613	Afumati	1	6	11	5
23.5634	46.0639	246	604335	Alba Iulia	4	2	5	3
25.3528	43.9779	75.5	359521	Alexandria	0	0	7	5
22.8595	44.9968	250	501252	Apa Neagra	0	1	3	0
21.3536	46.1335	116.6	608121	Arad	0	6	5	5
26.9125	46.5319	174	635658	Bacau	3	6	5	10
23.1131	44.4762	313	428307	Bacles	2	0	0	1
23.4916	47.6608	186.1	740330	Baia Mare	3	3	5	2
22.4164	44.881	190	452230	Baile Herculane	5	0	3	3
23.3312	44.0293	57	401321	Bailesti	0	1	4	1
23.3102	46.5355	1360	634322	Baisoara	5	1	1	1
24.6147	45.6039	2070	536437	Balea Lac	2	0	0	0
26.0782	44.5104	90	430608	Baneasa	2	5	11	7
21.1364	45.3827	83.4	523108	Banloc	3	2	6	1
25.5958	46.0808	508	605537	Baraolt	1	0	0	1
27.6444	46.233	172	614740	Barlad	6	4	7	8
23.9442	43.7897	36	347357	Bechet	2	1	1	0

Longitude (°)	Latitude (°)	Altitude (m.a.s.l.)	Station Id	Station Name	Breakpoints detected			
					PREC (mm)	Tavg (°C)	Tmin (°C)	Tmax (°C)
26.7103	45.5489	850	533642	Bisoca	3	0	0	0
24.5139	47.1491	366	708430	Bistrita	0	3	3	4
23.9352	46.1784	337	611355	Blaj	3	2	1	2
24.2715	45.653	518	538416	Boita	2	2	4	4
22.5902	46.9936	333	659236	Borod	3	1	1	2
26.6455	47.7356	161	741640	Botosani	1	1	3	1
22.0062	44.9183	256	455200	Bozovici	1	1	2	0
27.9197	45.2066	14.5	512755	Braila	0	6	8	5
25.5262	45.6958	534	542532	Brasov	2	2	5	1
25.2965	46.649	1282	639518	Bucin	0	0	0	0
26.8517	45.1327	97	509649	Buzau	3	6	5	13
22.946	43.9849	61	359257	Calafat	0	1	5	1
27.3383	44.2057	18.7	412721	Calarasi	2	4	6	3
25.2462	47.0979	2022	706515	Calimani	3	0	0	0
23.0404	46.3638	591	622303	Campeni	3	1	1	0
25.7334	45.1437	461	517545	Campina	1	2	7	2
25.0366	45.2748	680.7	517507	Campulung Muscel	2	7	6	5
24.3573	44.1001	106	406421	Caracal	0	6	5	6
22.2263	45.4171	241	525215	Caransebes	1	5	5	1
25.9499	46.9775	1897	656555	Ceahlau Toaca	1	0	0	0
28.0437	44.3456	87.2	421803	Cernavoda	0	6	6	1

Longitude (°)	Latitude (°)	Altitude (m.a.s.l.)	Station Id	Station Name	Breakpoints detected			
					PREC (mm)	Tavg (°C)	Tmin (°C)	Tmax (°C)
21.5417	46.5185	96	632130	Chisineu Cris	2	1	4	3
23.5713	46.7778	410	647334	Cluj-Napoca	4	9	4	6
28.6455	44.2138	12.8	413838	Constanta	1	3	3	2
28.342	44.7343	219.2	444820	Corugea	2	6	3	3
26.9256	47.3583	289	722657	Cot0ri	1	2	3	1
23.867	44.3101	192	414352	Craiova	5	7	15	6
22.5015	45.3005	1456	518231	Cuntu	6	0	1	0
24.6697	45.1788	448	509441	Curtea de Arges	1	1	2	6
26.5735	48.1949	259	812637	Darabani	1	0	0	0
23.8989	47.128	232	709352	Dej	2	1	1	0
22.8988	45.8649	240	553254	Deva	3	3	6	4
24.2372	44.6654	280	444417	Dragasani	2	6	4	7
22.6261	44.6265	77	438238	Drobeta Turnu-Severin	1	1	3	4
24.5916	46.2279	218	614436	Dumbraveni	1	1	4	2
22.1712	46.6446	586	639210	Dumbravita de Codru	1	0	0	3
24.9352	45.8362	428	551459	Fagaras	4	1	1	1
27.839	44.3915	58.3	422751	Fetesti	4	5	5	5
26.0938	44.4121	82	425606	Filaret	1	4	7	3
27.1998	45.6875	57	541712	Focsani	2	3	4	1
25.2715	45.4315	1384	528518	Fundata	0	1	1	0

Longitude (°)	Latitude (°)	Altitude (m.a.s.l.)	Station Id	Station Name	Breakpoints detected			
					PREC (mm)	Tavg (°C)	Tmin (°C)	Tmax (°C)
26.5236	44.4529	67	428632	Fundulea	2	0	0	0
28.0323	45.4729	69	530801	Galati	0	11	12	6
25.9327	43.8752	23.6	352557	Giurgiu	2	5	11	3
29.1568	45.1769	2.8	511912	Gorgova	4	3	3	0
27.2946	44.7408	50	445718	Grivita	2	6	5	7
28.9989	44.6898	2	441900	Gura Portitei	1	0	1	0
22.3333	46.2792	177	617220	Gurahont	3	1	4	3
27.9635	44.6917	37.5	441757	Harsova	3	3	10	2
22.1123	46.7886	163	646207	Holod	1	1	1	2
23.0325	46.8573	560	651305	Huedin	5	0	1	0
27.6283	47.1709	74.3	710736	Iasi	2	8	4	4
24.649	47.6026	1785	737439	Iezer	2	0	0	0
26.0568	45.6683	707	541601	Intorsura Buzaului	0	0	2	0
20.7024	45.7808	79	547042	Jimbolia	2	1	1	0
25.5126	46.7057	750	642540	Joseni	4	2	7	1
28.8764	44.7661	37.7	446853	Jurilovca	7	2	7	2
26.3755	45.8239	1776	551621	Lacauti	4	2	0	2
21.9333	45.6865	123	541154	Lugoj	3	1	2	3
29.0734	45.0872	167.5	505904	Mahmudia	1	0	2	0
28.5874	43.8161	6	349835	Mangalia	0	1	2	0
28.2514	44.2432	69.5	415816	Medgidia	4	3	0	3
25.7726	46.3713	661	622544	Miercurea Ciuc	4	1	3	1

Longitude (°)	Latitude (°)	Altitude (m.a.s.l.)	Station Id	Station Name	Breakpoints detected			
					PREC (mm)	Tavg (°C)	Tmin (°C)	Tmax (°C)
21.6333	44.7224	82	444127	Moldova Veche	2	1	1	1
24.5701	45.0163	548	500432	Moraresti	0	0	1	0
27.4421	46.8381	133	650727	Negresti	0	1	1	0
23.6308	45.4355	1348	526338	Obarsia Lotrului	5	1	0	0
23.9405	47.777	503	747356	Ocna Sugatag	0	1	2	4
25.2917	46.2968	523	618518	Odorheiul Secuiesc	4	0	2	3
26.6371	44.0747	14.9	404638	Oltenita	0	1	1	2
21.8959	47.0357	136	703156	Oradea	0	5	8	4
21.7105	45.0387	309	502141	Oravita	2	3	4	4
23.9324	45.6571	1453	539357	Paltinis	1	0	0	1
23.4631	45.3874	1548	523328	Parang	1	0	0	2
26.3695	45.3246	289	519622	Patarlagele	0	2	1	1
26.4098	45.6027	1632	536625	Penteleu	2	1	2	0
23.3767	45.4063	607	525323	Petrosani	4	2	4	3
26.3895	46.9337	360	656621	Piatra Neamt	1	2	4	5
24.866	44.8489	316	452452	Pitesti	5	2	6	5
25.9874	44.9557	177	457600	Ploiesti	2	3	6	4
25.1344	47.3246	923	719507	Poiana Stampei	3	0	1	3
23.8086	45.1654	531	511349	Polovragi	3	4	7	4
25.5835	45.5063	1090	530535	Predeal	3	1	4	4

Longitude (°)	Latitude (°)	Altitude (m.a.s.l.)	Station Id	Station Name	Breakpoints detected			
					PREC (mm)	Tavg (°C)	Tmin (°C)	Tmax (°C)
25.8904	47.8378	389	751555	Radauti	4	0	0	0
27.0385	45.3906	152	523703	Ramnicu Sarat	5	0	5	2
24.3628	45.0888	237	506422	Ramnicu Valcea	0	4	4	4
21.887	45.3144	279	518155	Resita	1	1	1	0
26.9118	46.9691	216	655650	Roman	4	3	3	6
23.139	46.3176	1196	619308	Rosia Montana	0	0	0	1
24.9787	44.1072	102.2	407500	Rosiori de Vede	5	5	3	5
22.0945	47.3441	124	722205	Sacuieni	3	1	3	2
20.6016	46.0713	85	604037	Sannicolau Mare	1	3	2	4
24.1598	46.7475	399	645410	Sarmasu	2	0	0	1
22.8872	47.7215	123	748253	Satu Mare	2	1	2	2
23.5415	45.9641	271	557334	Sebes	2	1	2	1
22.0558	45.1814	1432	507158	Semenic	4	0	1	0
29.5991	44.8976	1.4	454936	Sf. Gheorghe Delta	3	0	3	2
25.8021	45.8715	523	552548	Sf. Gheorghe Munte	3	3	5	4
25.5142	45.355	1510	523530	Si0ia 1500	3	1	5	2
24.0914	45.7893	443	548409	Sibiu	1	5	6	6
23.9043	47.9393	275	758355	Sighetul Marmatiei	3	1	1	3

Longitude (°)	Latitude (°)	Altitude (m.a.s.l.)	Station Id	Station Name	Breakpoints detected			
					PREC (mm)	Tavg (°C)	Tmin (°C)	Tmax (°C)
24.3545	44.4422	172	426421	Slatina	1	1	3	2
27.3835	44.5528	51.2	433724	Slobozia	3	3	4	2
22.6234	46.6898	1108	641237	Stana de Vale	4	1	2	4
27.2197	47.8322	110	749713	Stanca Stefanesti	1	0	1	0
22.4665	46.528	278	632229	Stei	4	1	6	2
24.7898	44.5627	208.7	436447	Stolnici	1	2	4	3
26.2405	47.6329	352	739615	Suceava	1	0	3	0
29.7589	45.1485	12.7	509940	Sulina	2	1	5	2
22.7836	47.4551	159	728247	Supuru de Jos	1	0	0	1
24.226	46.3601	523	622414	Tarnaveni	1	2	1	3
22.5327	45.281	2180	515231	Tarcu	7	0	0	0
25.4257	44.9295	293.5	456526	Targoviste	0	3	6	4
22.7262	46.1695	273	610244	Tebea	5	1	1	0
27.409	45.8416	60	551716	Tecuci	6	5	4	7
23.2593	45.0406	204.3	502317	Tg. Jiu	1	1	2	5
23.8722	47.4396	363	726352	Tg. Lapus	0	0	2	2
23.7087	44.8781	262	453344	Tg. Logresti	0	1	2	0
24.5338	46.5333	310	632432	Tg. Mures	4	3	5	3
26.3792	47.2121	387	714623	Tg. Neamt	1	3	7	1
26.641	46.2727	242	617637	Tg. Ocna	1	1	1	1
26.1151	45.9929	568	600608	Tg. Seciuesc	0	0	2	0

Longitude (°)	Latitude (°)	Altitude (m.a.s.l.)	Station Id	Station Name	Breakpoints detected			
					PREC (mm)	Tavg (°C)	Tmin (°C)	Tmax (°C)
21.2581	45.7711	86	546115	Timisoara	1	6	2	7
25.5792	44.6529	159	439534	Titu	0	0	2	3
25.3599	46.9264	687	655522	Toplita	2	0	0	2
28.8241	45.1905	4.4	511849	Tulcea	0	2	5	1
23.7912	46.583	427	635347	Turda	2	1	4	4
24.8785	43.7602	30.6	346452	Turnu Magurele	3	4	3	2
26.6572	44.7217	60	443639	Urziceni	4	2	5	3
22.151	46.0192	156	602213	Varadia de Mures	1	1	2	1
27.7144	46.6461	116	639744	Vaslui	2	1	3	4
25.4567	45.4458	2504	527527	Vf. Omu	8	0	0	2
25.537	44.2829	106.2	417530	Videle	0	4	3	3
22.7942	46.7592	1836	646247	Vladeasa 1800	2	0	0	1
23.967	45.4112	573	525358	Voineasa	1	0	0	0
23.0467	47.1949	295	711305	Zalau	5	0	5	2
25.3536	43.6615	33.6	340521	Zimnicea	2	1	3	0

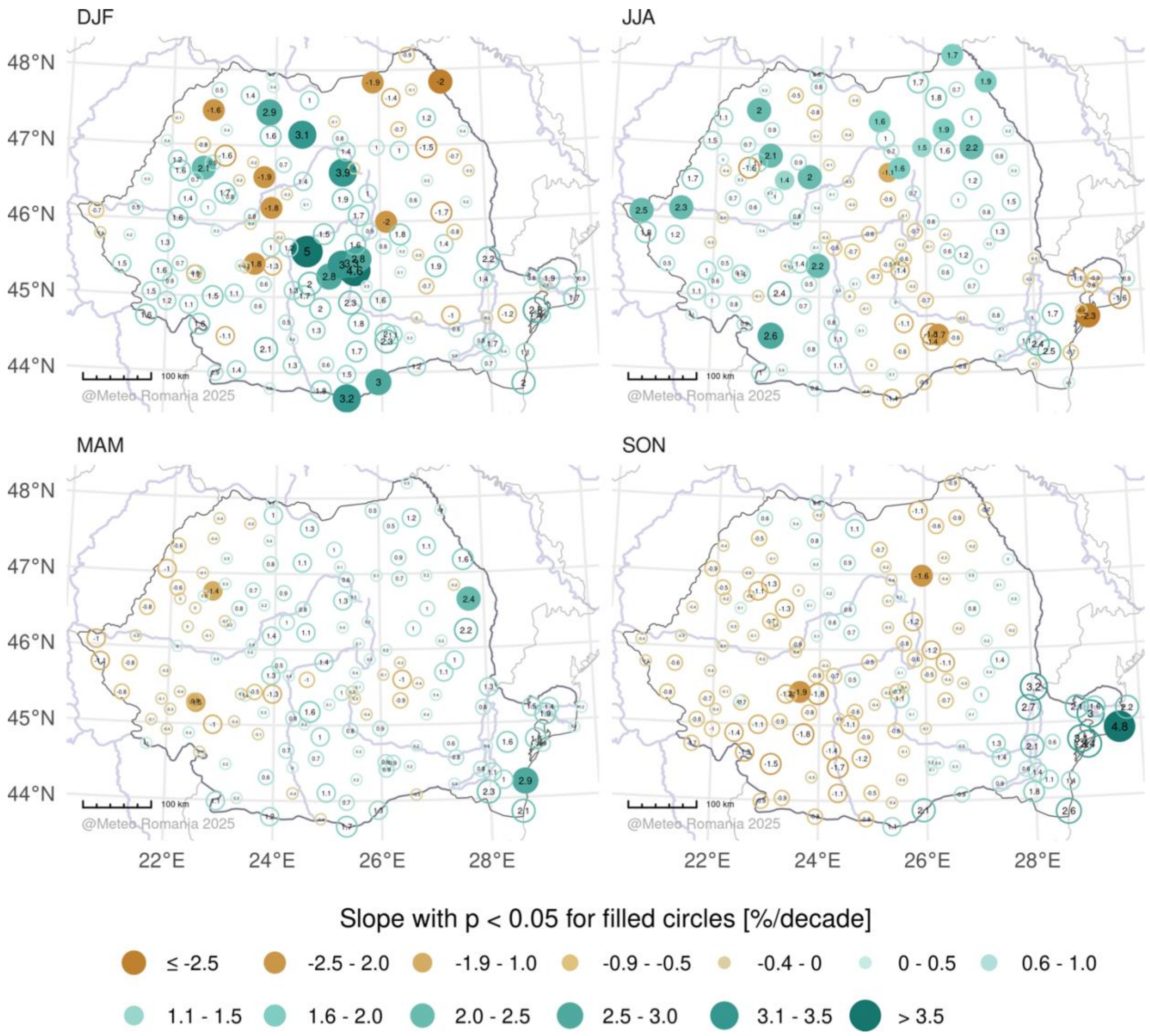

Fig. S1 Seasonal Theil–Sen slopes and Mann-Kendall significance levels (filled circles) evaluated on homogenized yearly time series of precipitation (%/decade).

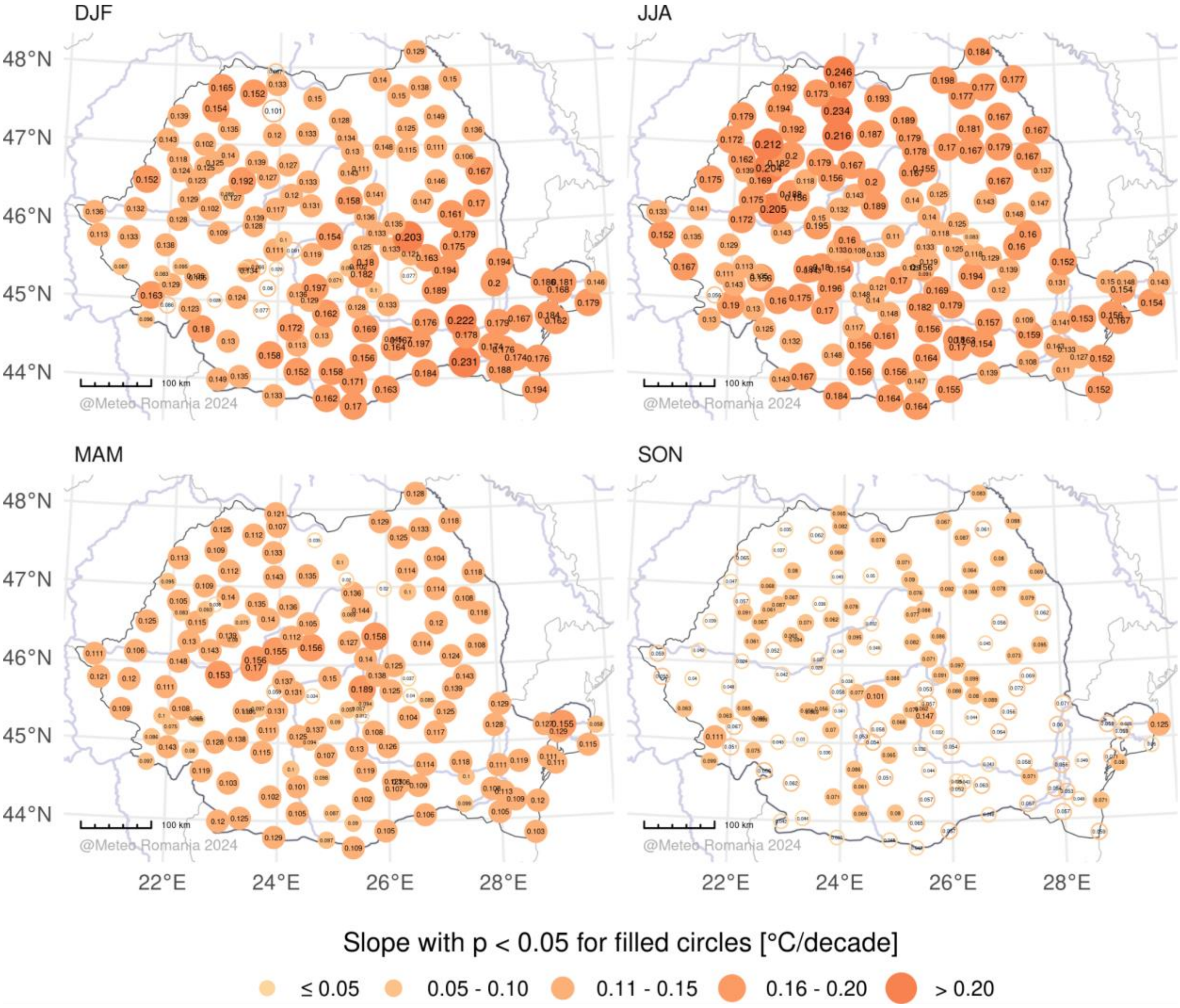

Fig. S2 Seasonal Theil–Sen slopes and Mann-Kendall significance levels (filled circles) evaluated on homogenized yearly time series of average air temperature (°C/decade).

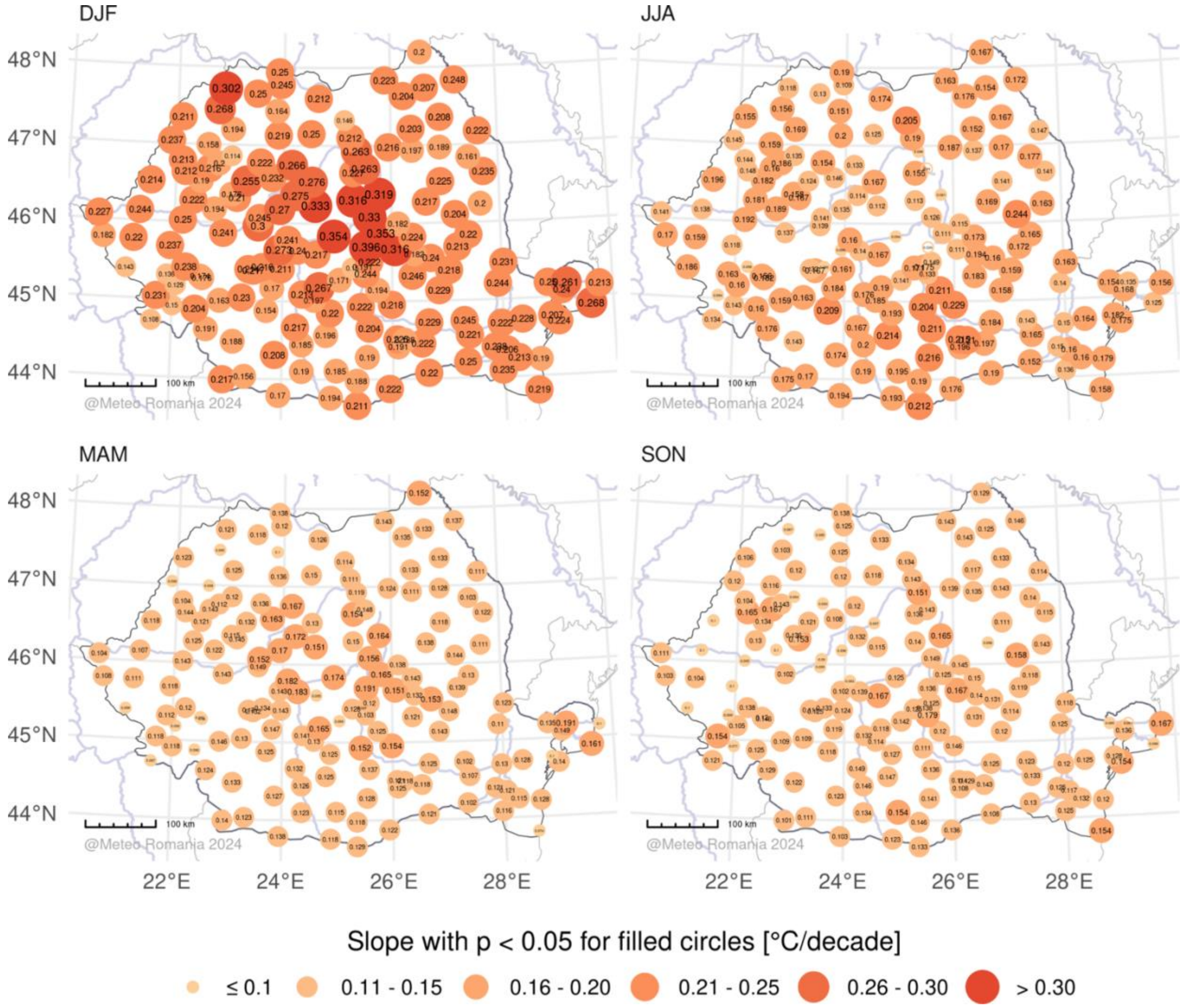

Fig. S3 Seasonal Theil–Sen slopes and Mann-Kendall significance levels (filled circles) evaluated on homogenized yearly time series of minimum air temperature (°C/decade).

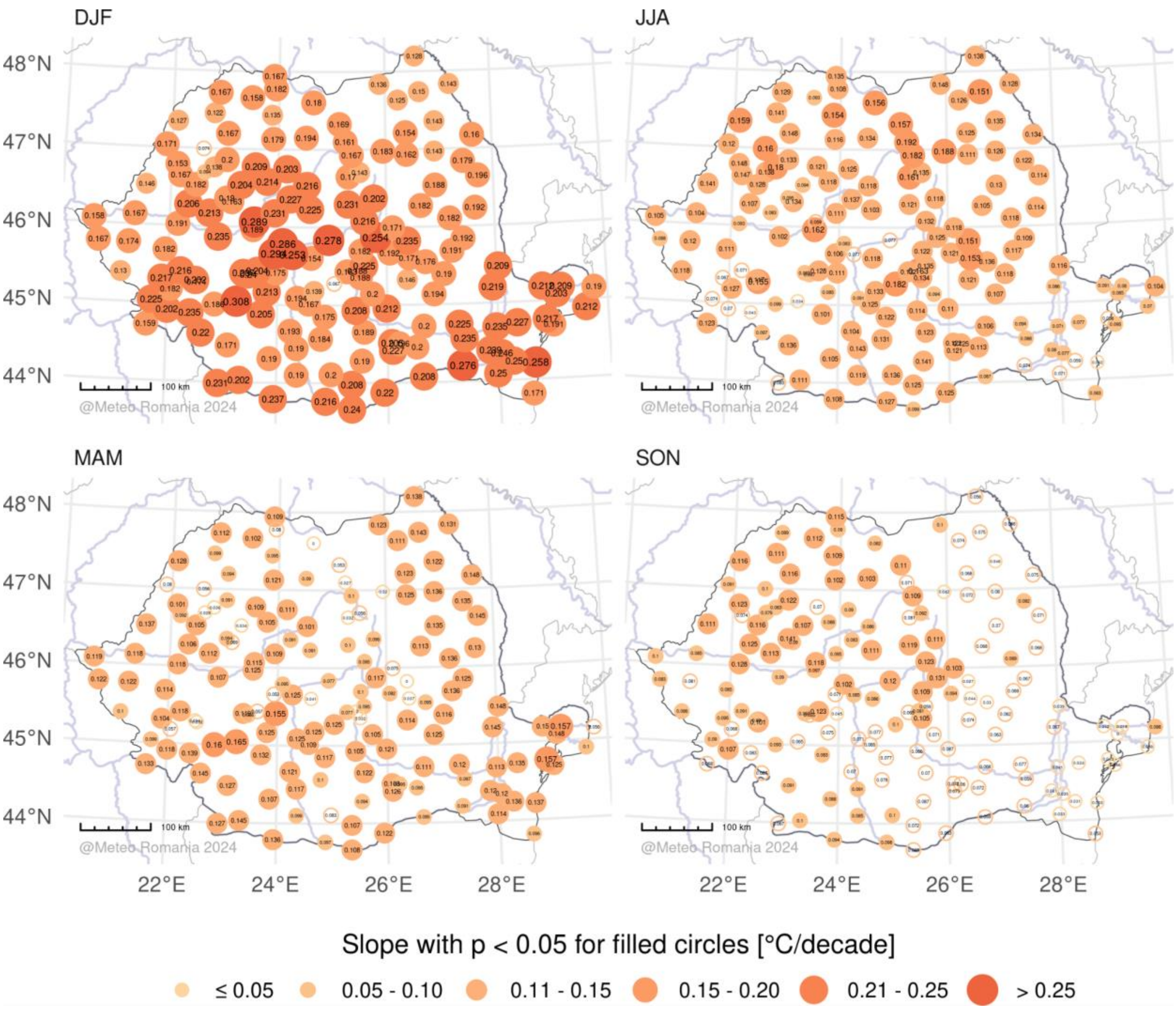

Fig. S4 Seasonal Theil–Sen slopes and Mann-Kendall significance levels (filled circles) evaluated on homogenized yearly time series of maximum air temperature (°C/decade).

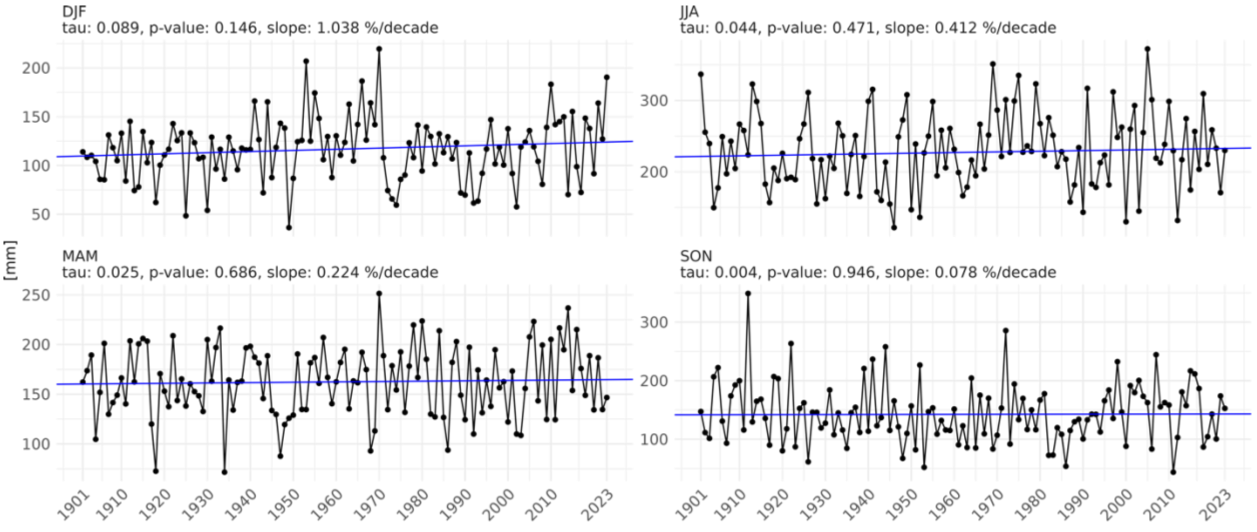

Fig. S5 Trends at the country level for seasonal precipitation computed from the homogenized dataset. Mann-Kendall tau, p-value, and Theil-Sen slopes quantitatively measure the trends' significance and magnitude.

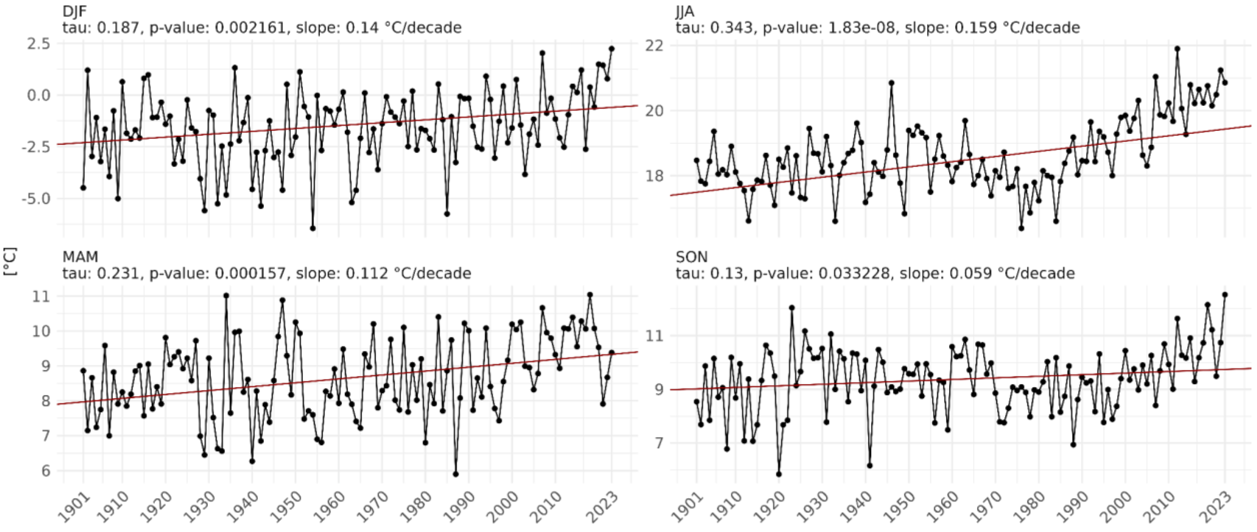

Fig. S6. Trends at the country level for seasonal average air temperature computed from the homogenized dataset. Mann-Kendall tau, p-value, and Theil-Sen slopes quantitatively measure the trends' significance and magnitude.

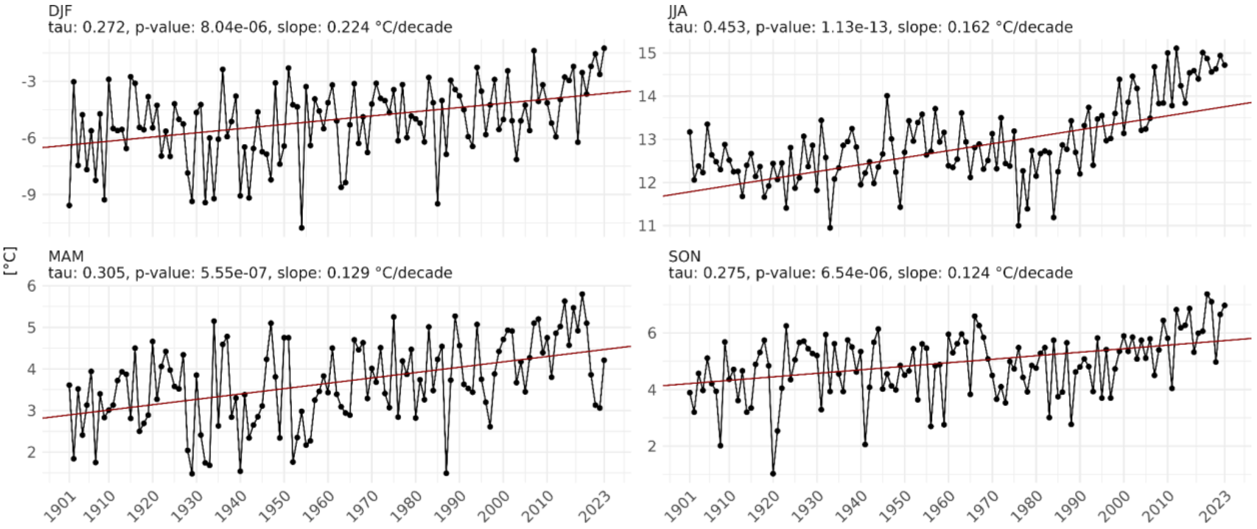

Fig S7 Trends at the country level for seasonal minimum air temperature computed from the homogenized dataset. Mann-Kendall tau, p-value, and Theil-Sen slopes quantitatively measure the trends' significance and magnitude.

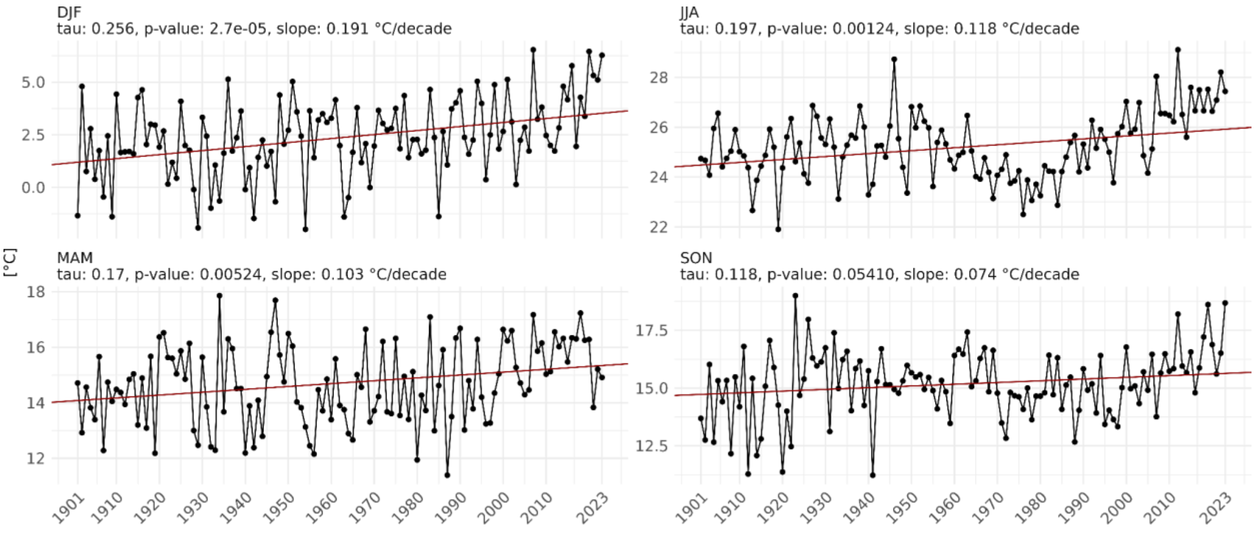

Fig. S8 Trends at the country level for seasonal maximum air temperature computed from the homogenized dataset. Mann-Kendall tau, p-value, and Theil-Sen slopes quantitatively measure the trends' significance and magnitude.
